# EMOKINE: A software package and computational framework for scaling up the creation of highly controlled emotional full-body movement datasets

**DOI:** 10.3758/s13428-024-02433-0

**Published:** 2024-06-25

**Authors:** Julia F. Christensen, Andrés Fernández, Rebecca A. Smith, Georgios Michalareas, Sina H. N. Yazdi, Fahima Farahi, Eva-Madeleine Schmidt, Nasimeh Bahmanian, Gemma Roig

**Affiliations:** 1 Department of Cognitive Neuropsychology, Max Planck Institute for Empirical Aesthetics, Frankfurt/M, Germany; 2 Methods of Machine Learning, University of Tübingen, Tübingen, Germany; 3 International Max Planck Research School for Intelligent Systems, Tübingen, Germany; 4 Department of Psychology, University of Glasgow, Glasgow, Scotland; 5 WiseWorld.AI, Porto, Portugal; 6 Max Planck School of Cognition, Leipzig, Germany; 7 Department of Language and Literature, Max Planck Institute for Empirical Aesthetics, Frankfurt/M, Germany; 8 Department of Modern Languages, Goethe University, Frankfurt/M, Germany; 9 Computer Science Department, Goethe University, Frankfurt/M, Germany; 10 The Hessian Center for Artificial Intelligence (hessian.AI), Darmstadt, Germany; 11 Center for Humans and Machines, Max Planck Institute for Human Development, Berlin, Germany

**Keywords:** Emotion, Motion capture, Computer vision, Affective neuroscience, Aesthetics, Dance, Dataset, Open science

## Abstract

EMOKINE is a software package and dataset creation suite for emotional full-body movement research in experimental psychology, affective neuroscience, and computer vision. A computational framework, comprehensive instructions, a pilot dataset, observer ratings, and kinematic feature extraction code are provided to facilitate future dataset creations at scale. In addition, the EMOKINE framework outlines how complex sequences of movements may advance emotion research. Traditionally, often emotional-‘action’-based stimuli are used in such research, like hand-waving or walking motions. Here instead, a pilot dataset is provided with short dance choreographies, repeated several times by a dancer who expressed different emotional intentions at each repetition: anger, contentment, fear, joy, neutrality, and sadness. The dataset was simultaneously filmed professionally, and recorded using XSENS® motion capture technology (17 sensors, 240 frames/second). Thirty-two statistics from 12 kinematic features were extracted offline, for the first time in one single dataset: speed, acceleration, angular speed, angular acceleration, limb contraction, distance to center of mass, quantity of motion, dimensionless jerk (integral), head angle (with regards to vertical axis and to back), and space (convex hull 2D and 3D). Average, median absolute deviation (MAD), and maximum value were computed as applicable. The EMOKINE software is appliable to other motion-capture systems and is openly available on the Zenodo Repository. Releases on GitHub include: (i) the code to extract the 32 statistics, (ii) a rigging plugin for Python for MVNX file-conversion to Blender format (MVNX=output file XSENS® system), and (iii) a Python-script-powered custom software to assist with blurring faces; latter two under GPLv3 licenses.

## Summary & background

### Summary

EMOKINE is a software and dataset creation framework for highly controlled kinematic datasets of emotionally expressive full-body movements. The primary novelty of this framework is that it provides the research community with a set of 12 readily computed kinematic features (32 statistics in total) that can be used out-of-the-box by researchers in order to model emotional expressivity in full-body movement. For the first time, these 12 features are presented together: speed, acceleration, angular speed, angular acceleration, limb contraction, distance to center of mass, quantity of motion, dimensionless jerk (integral), head angle (with regards to vertical axis and to back), and space (convex hull 2D and 3D). A pilot dataset accompanies this article, of realistic full-body movement stimuli, which have been designed and instantiated with all other parameters controlled so that most of the variability stems from the different expressed emotions. The pilot data for EMOKINE were recorded via the XSENS® system, however, the software is also appliable to data obtained from other motion-capture systems with minimal to no changes.

Based on the creation procedure of this pilot dataset, we describe a process by which such a dataset creation can be scaled up in the future, while ensuring mandatory levels of experimental control that are key for datasets to be used in experimental psychology and affective neuroscience with human participants (Christensen & Calvo-Merino, 2013; Christensen & Jola, 2015), and ensuring the technical detail required for datasets in computer vision and related disciplines. Importantly, instead of following the model of traditional datasets in emotion science that comprise emotional-‘action’-based stimuli, like hand-waving or walking motions, the EMOKINE pilot dataset contains complex sequences of movements: 6-s-long dance choreographies. A dancer choreographed a series of short, simple ballet-movement-inspired dance sequences of approximately 6 s in length, which corresponds to eight counts in dance notation. Then she performed the sequences six times each, expressing a different emotional intention at each repetition – namely anger, contentment, fear, joy, neutrality, and sadness. Classically, most datasets include only the ‘basic’ emotions proposed by Paul Ekman and colleagues (Ekman, 1973/2015; Ekman & Friesen, 1971) – namely, anger, fear, joy, neutrality, and sadness. We extended the spectrum of expressed emotions in the EMOKINE pilot dataset by also adding the emotion ‘contentment’, which is another positive-valence emotion like joy, but of low arousal; symmetrical to what anger (negative valence, high arousal) is to sadness (negative valence, low arousal).

The EMOKINE suite includes:

- a detailed step-by-step procedure guide to create EMOKINE datasets at scale;
- a pilot dataset (recorded with the XSENS® system) with four different visual presentations for each video stimulus – namely (i) avatars, (ii) full-light displays (FLDs) with blurred face, (iii) point-light displays (PLDs), and (iv) silhouettes;
- the raw kinematic data for each stimulus of the pilot dataset, obtained via the XSENS® motion capture (LINK) system (via 17 sensors distributed on the body, recorded at 240 frames/second);
- the code to obtain 32 statistics from the 12 kinematic features;
- human observers’ emotion recognition rates and value judgments, which confirm the intended emotional categories of the pilot dataset.

The pilot dataset of EMOKINE is available for download on the Zenodo repository and the software on GitHub. Releases on GitHub include:

- an extensive repository of code to extract the 32 statistics of the 12 kinematic features – namely, speed, acceleration, angular speed, angular acceleration, limb contraction, distance to center of mass, quantity of motion, dimensionless jerk (integral), head angle (with regards to vertical axis and to back), and space (convex hull 2D and 3D). Average, median absolute deviation (MAD) and maximum value were computed for each;
- A MVNX rigging plugin for Python that allows Blender to convert MVNX files to a Blender-friendly format (MVNX = output file of the motion capture XSENS system);
- Python-script-powered custom software to assist with the process of blurring faces. The latter two have been released under GPLv3 licenses, and all are available for download on GitHub (see data availability statement, Section 11).

The GitHub readme file includes an explanation on how to apply the EMOKINE software to data obtained from other systems. In particular, points (a) and (c) can be applied to any data including keypoint positions with minimal to no change. Point (b) naturally depends on the MVNX format (which is given by the XSENS system), but it can be ignored for other formats.

## Background

Investigating emotion recognition competence is important due to its significance for psychosocial functioning (Cosmides & Tooby, 2000; Darwin, 1872/2009; Ekman, 1973/2015; Ekman & Friesen, 1971). In the fields of emotion psychology, affective neuroscience, and computer vision, such research often relies on datasets that comprise stimuli of pictures or videos of facial and bodily expressions of emotions. Yet, compared to the extensive existing research on the recognition of emotions from facial expressions (Byron et al., 2007; Elfenbein & Ambady, 2002; O'Boyle Jr et al., 2011; Rosete & Ciarrochi, 2005; Rubin et al., 2005; Scherer & Scherer, 2011; Walter et al., 2012; Zuskin et al., 2007), the bodily channel of emotional expression has received less empirical attention, despite important calls to extend emotion perception research to this domain (Aviezer et al., 2012; Bellot et al., 2021; de Gelder, 2006, 2009; Keck et al., 2022; McCarty et al., 2017; Vaessen et al., 2018). Less stimulus materials are available, and available datasets suffer from limitations (discussed in, e.g., Christensen & Calvo-Merino, 2013; Christensen & Jola, 2015; Smith & Cross, 2022). Besides, most full-body datasets of emotional expressions show actors performing different emotional actions; for instance, punching a fist in anger, sinking to the floor in sadness, jumping with joy (Atkinson et al., 2004; Crane & Gross, 2013; Dael et al., 2012; Gross et al., 2010). However, this approach likely measures the ability to recognize familiar prototypical *actions* indicative of different emotions, rather than perceptual sensitivity to emotional expressions (Shafir, 2016; Shafir et al., 2013). In order to avoid such confounding effects and investigate the manifestation of different emotions in one same human movement, a same-sequence approach can be a valid alternative, where a set of different movements are repeated several times, with each repetition corresponding to the expression of a different emotion. In other words, the expressor in every repetition performs the exact same reference movements, with the only source of intentional variability in the kinematics being due to the intended expression of a different emotion.

Therefore, walking, pointing, drinking, knocking, or throwing movements have recently been proposed as a valid movement alternative for emotion recognition and emotion perception research (Crane & Gross, 2007; Dekeyser et al., 2002; Heberlein et al., 2004; Krüger et al., 2018; Ma et al., 2006; Pollick et al., 2001; Roether et al., 2009; Vanrie & Verfaillie, 2004). Yet, such movements are rather simple and may be confounded with stereotypical assumptions about these movements. For example, in everyday life, walking patterns are typically associated with how much someone is rushing or with the existence of injuries. With the objective of reducing contextual cues from movement stimulus materials, and movement towards more varied patterns of movements, dance movements have recently been proposed as stimulus materials. Dance is, par excellence, an instance of emotionally expressive full-body movement (Boone and Cunningham, 1998; Boone and Cunningham, 2001; Christensen et al., 2019; Kirsch et al., 2016; Dittrich et al., 1996; Orgs et al., 2016; Van Dyck et al., 2017; Van Dyck et al., 2013; Van Meel et al., 1993). Dance choreographies can be created to be more varied than simple walking or throwing movements. Besides, compared to walking and throwing movements, using choreographies that are novel to participants in emotion perception research reduces possible familiarity effects. Besides, professional dancers are trained to express different emotional states with one same dance gesture and can therefore serve as models for stimulus materials (Christensen et al., 2019; Karin, 2016; Karin et al., 2016). It is relevant to note that we are not proposing that same-sequence stimuli should completely replace emotional-action-based stimuli in emotion research. We propose that these options are alternative stimuli materials and should be chosen depending on the research question. Here we focus on the advantage of same-sequence stimuli materials.

This *same-sequence-different*-emotional expressivity in dance movements is comparable to language. The same sentence can be spoken to sound either angry or happy to a listener, depending on how the expressor modulates their voice with their breathing and the muscles of their vocal tract (Bänziger et al., 2009; Scherer & Scherer, 2011; Scherer et al., 2017). How a dancer performs one same dance movement sequence, at several repetitions, with different emotional intentions, has previously been found to convey these intended emotional states to human observers, even to those without dance experience (Christensen et al., 2017; Christensen et al., 2019; Christensen et al., 2014; Christensen et al., 2023). In classical emotion-recognition tasks with dance movements, average recognition rates are, generally, above chance level, and vary between 18.04% (Christensen et al., major revisions), and 48% (Christensen et al., 2023; Smith & Cross, 2022).

With the advances of filming and motion-capture technologies of the past decades, new horizons have opened up for the digitization and analysis of full-body movement.

By combining these recent technologies with the same-sequence-different-emotion approach, the EMOKINE framework offers a novel route for emotion perception research with full-body movement. The stimuli design is based on previous datasets of dance movements that did, however, not include motion-capture technology (Christensen et al., 2023; Christensen et al., 2017; Christensen et al., major revisions; Christensen et al., 2019; Christensen et al., 2014; Smith & Cross, 2022).

## Objectives

We had four objectives with EMOKINE:

1. To create a *pilot* dataset of simple dance movement sequences, with each sequence performed by a dancer six times, each with a different intended emotion out of a pool of six possible emotions – namely anger, contentment, fear, joy, neutrality, and sadness. The novelty of this work is that this same-sequence–different-emotion experimental design was complemented by time-resolved, whole-body kinematics data, measured by motion-capture technology. Please note that this dataset was created to enable the development of the EMOKINE suite and contains portrayals from only one dancer. To ensure generalizability in future research using the EMOKINE software, datasets should include portrayals from more than one dancer.
2. To render these pilot stimuli in four visual presentations for further study: (i) videos showing an avatar performing the movements, extracted from the XSENS propriety software (avatar videos); (ii) videos showing the dancer in full light, but with blurred face, to avoid emotion recognition from the face (full-light displays; FLDs); (iii) videos showing point-light displays (PLDs), which have been rendered with a Blender-based algorithm (Blender Community, 2018); and (iv) videos showing black-and-white silhouettes, where the movement has been extracted from a greenscreen background to show only a white silhouette moving in front of a black background (silhouette videos).
3. To compute a total of 32 statistics of 12 kinematic features, for the first time, in one single dataset with same-sequence stimuli materials and make the software freely available.
4. To obtain emotion-recognition judgments and beauty ratings from human participants for all the created stimuli mentioned above. The emotion-recognition judgments were obtained to validate the pilot dataset in terms of the intended emotional expression of the dancer. Beauty judgments were obtained to encourage the use of aesthetic judgment as an implicit emotion recognition task with future datasets. Previous research has shown that while emotion-recognition rates for a stimulus set may be low, significant differences can usually be found between stimuli intended to express different emotions (Christensen et al., 2023; Christensen et al., 2019), making aesthetic judgment (e.g., beauty, liking, etc.) an interesting implicit emotion-recognition task for the future.

## Method

Ethical approval for the experiment was in place through the Umbrella Ethics approved by the Ethics Council of the Max Planck Society (Nr. 2017_12). For the observer experiment (performed online via the platform Prolific®; Peer et al., 2021; Stanton et al., 2022), informed consent was obtained from all participants, and was given online through a tick-box system. All methods were performed in accordance with the relevant guidelines and regulations.

### Open science statement

In accordance with the framework for open and reproducible science (Munafò et al., 2017), all measures that we collected in the study are reported here.

### Participants

#### Participant pilot dataset creation (one dancer)

One female former professional dancer with 20 years of ballet dance experience choreographed and performed the dance movement sequences.

#### Participants’ online experiment

In total, 172 participants (57 male, one other) participated in the human emotion-recognition task (mean age = 35.89 years, *SD* = 11.93, range, 18–65). From the original sample, 22 participants were excluded due to technical issues (the video stimuli did not play), or for not passing attention checks (on two of the emotion-recognition trials, cartoon videos were shown with very obvious emotional expressions (Sponge Bob crying a river of tears; correct response: sad; and Mikey Mouse’s head turning red and exploding; correct response: angry). The experiment took approximately 50 min and participants were paid via the Prolific platform (£8/h). Participants had an average of 1.5 years of hobby dance experience (*SD* = 5.05, range, 0–40). We had set the Prolific® filter to return only participants whose first language was English, to ensure complete comprehension of study instructions.

To determine the sample size, we used G*Power 3.1. (Faul et al., 2007). Because the stimuli of the EMOKINE dataset were presented to participants in four different types of visual presentations (avatars, full-light displays (FLDs), point-light displays (PLDs), silhouettes), there was a total of 216 stimuli. Rating this many stimuli could have led to participant fatigue. To avoid this, we opted for dividing stimuli randomly into four sets and determined the sample size for each group of participants for these four sets of stimuli. Subsequently, we confirmed that the percentage of correct responses given by participants in these four different groups to the stimuli was equivalent. We choose a threshold for a large effect size of *d* = .80 (Cohen, 1988) because large effect sizes indicate that the research finding has practical significance. We had initially planned to compare the percentage of correct responses between the four groups with an independent *t* test. As a result, the suggested sample size calculation for independent samples *t* test (effect size = .80; alpha = .05; power = .90) was 28 per group. However, we tested at least 30 participants on each of the four sets to ensure full randomization (30 can be divided by six emotions, 28 cannot). Due to technical difficulties, the final number of participants in each group was; group 1: *N* = 36; group 2: *N* = 32; group 3: *N* = 33; group 4: *N* = 31.

### Materials

#### Hardware

Motion capture was performed by means of the MVN Link system (XSENS®, 2020, 2023). Motion capture in this context is the act of recording the motion through time of a set of landmarks (also called keypoints) that are representative of a full-body human pose. This technology has matured mainly via two different approaches: optical solutions, in which markers on the body allow to locate the keypoints, and inertial/magnetic solutions, in which a set of sensors is placed on the body. Both approaches have advantages and disadvantages, but it is generally understood that while optical systems provide very high positional precision, inertial systems are more robust, versatile, and provide more stable acceleration readings (Lim et al., 2016; Skogstad et al., 2011). We are using the latter method in the current research.

The XSENS® system combines inertial and magnetic sensors with advanced algorithms and biomechanical models to provide highly reliable and accurate readings with high spatio-temporal resolution (Schepers & Giuberti, 2018). This allows an optimal assessment of kinematic parameters for complex movement such as dance.

In its full-body configuration, the MVN Link system provides kinematic information from 23 keypoints via a set of 17 wireless sensors embedded on different parts of a spandex suit that fits the dancer’s body. This setup is designed to allow for highly free and complex motion.

**Fig. 1:**
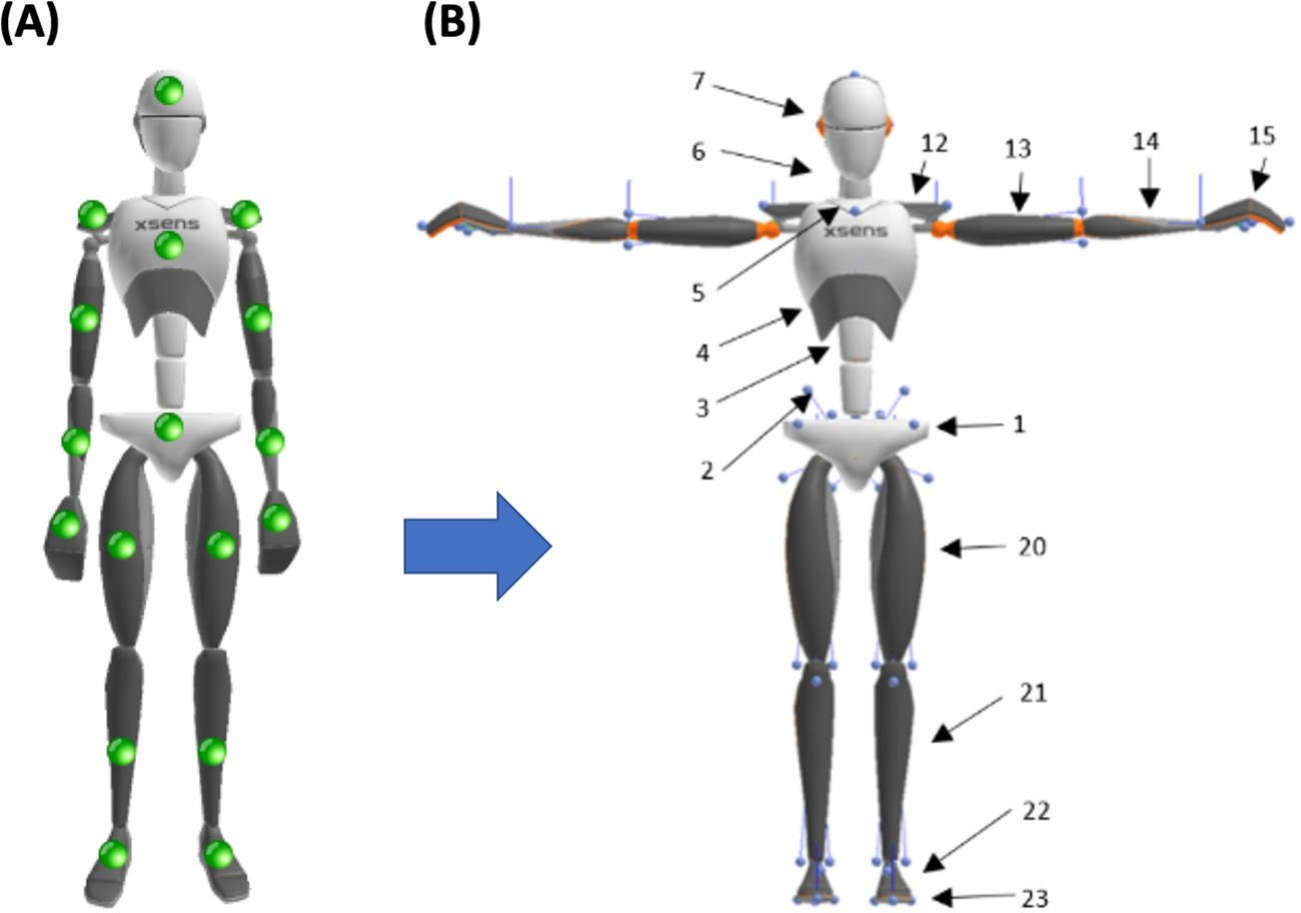
Sensors and segments (keypoints) of the XSENS MVN biomechanical model. *Note*: **(A)** The *large green spheres* on the model illustrate the location of the 17 sensors embedded in the spandex suit of the XSENS Link System. **(B)** The *small blue spheres* on the model illustrate the joints that are used to compute the 23 segment (keypoint) values, shown with *arrows* and *numbers*, via the biomechanical model of the XSENS software. Reproduced with permission from the XSENS fact sheet about the biomechanical model; XSENS, 2023)

The keypoints are called “segments” in the XSENS Manual (see Sections 7.2.5 and 15.4 for more details; XSENS Manual, 2020). For an overview of the 17 sensors and how they translate into information about the 23 keypoints, see Table 1 and Fig. 1 (reproduced from the XSENS manual, Section 15.4. (XSENS Manual, 2020), and the XSENS fact sheet about the biomechanical model; XSENS, 2023).

**Table 1:** The 23 body keypoints provided by the XSENS® software

Keypoint number	Segment Label (Bone)	Description	Joint
1	Pelvis	Segment between both hip joints and joint L5S1	jL5S1
2	L5	Segment between joints jL5S1 and jL4L3	jL4L3
3	L3	Segment between joints jL4L3 and jL1T12	jL1T12
4	T12	Segment between joints jL1T12 and jT9T8	jT9T8
5	T8	Segment between joints jT9T8 and jT1C7	jT1C7
6	Neck	Segment between joints jT1C7 and jC1Head	jC1Head
7	Head	End segment above joint jC1Head	jRightC7Shoulder
8	Right shoulder	Segment between joints jRightC7Shoulder and jRightUpperArm GH	jRightShoulder
9	Right upper arm	Segment between joints jRightUpperArm GH and jRightElbow	jRightElbow
10	Right forearm	Segment between joints jRightElbow and jRightWrist	jRightWrist
11	Right hand	End segment after joint jRightWrist	jLeftC7Shoulder
12	Left shoulder	Segment between joints jLeftC7Shoulder and jLeftUpperArm GH	jLeftShoulder
13	Left upper arm	Segment between joints jLeftUpperArm GH and jLeftElbow	jLeftElbow
14	Left forearm	Segment between joints jLeftElbow and jLeftWrist	jLeftWrist
15	Left hand	End segment after joint jLeftWrist.	jRightHip
16	Right upper leg	Segment between joints jRightHip and jRightKnee	jRightKnee
17	Right lower leg	Segment between joints jRightKnee and jRightAnkle	jRightAnkle
18	Right foot	Segment between joints jRightAnkle and jRightToe	jRightBallFoot
19	Right toe	End segment after joint jRightToe.	jLeftHip
20	Left upper leg	Segment between joints jLeftHip and jLeftKnee	jLeftKnee
21	Left lower leg	Segment between joints jLeftKnee and jLeftAnkle	jLeftAnkle
22	Left foot	Segment between joints jLeftAnkle and jLeftToe	jLeftBallFoot
23	Left toe	End segment after joint jLeftToe.	

Overview of the keypoints that were provided by the XSENS system and were used in subsequent analyses. This table has been reproduced from the XSENS manual, Section 15.4. In its “full-body” configuration, the MVN Link system from XSENS provides information for 23 keypoints (called “segments” in the XSENS Manual, see Sections 7.2.5 and 15.4; XSENS Manual, 2020), based on the computations via the 17 sensors, placed throughout the body. Reproduced with permission from the XSENS manual, Section 15.4. (XSENS Manual, 2020), and the XSENS fact sheet about the biomechanical model (XSENS, 2023)

The XSENS® recording and filming took place in the ArtLab foyer of the Max Planck Institute for Empirical Aesthetics in Frankfurt am Main, Germany, in front of a standard 6 × 3m chroma-key greenscreen background (LTT Junior Truss system with Premium green Buehnenmolton). This allowed for the creation of additional visual preparations of the stimuli, such as silhouette videos. For this, dedo-stage lights (AX3 light drop; 15W RGBW CREE LED) were used to illuminate the entire greenscreen and to minimize shadows.

To produce additional visual presentations of the dataset (FLDs and silhouettes), the dancer was also filmed using a camera Canon EOS 5D Mark IC camera with a Canon EF 24–105 mm f/4 L IS USM lens (settings: e.g., framerate (raw) at 50 fps and framerate (output) at 25 fps. White balance: 5000k, shutter speed: 1/100 sec, and ISO: 400. Video format: H.264, aspect ratio: 16:9, and resolution: 1920 × 1080).

Postproduction of the video footage was done on a 15-inch MAC Book Pro (2017; Processor: 2.9-GHz Quad-Core Intel Core i7; Memory: 16 GB 2133-MHz LPDDR3; Graphics: Radeon Pro 560 4 GB; Intel HD Graphics 630 1536 MB.

#### Software

The XSENS® company provides proprietary software that allows calibrating and monitoring the setup during recordings, as well as reading the sensors with a framerate of up to 240 Hz, and also the ability to edit and export the recorded data in various formats including video, and to MVNX (a form of XML; see the XSENS Manual (2020), chapters 6 to 10, for more details). The MVNX provides raw sensor data, as well as refined readings for positions, accelerations, and angles of the full-body keypoints (for more details, see the XSENS Manual, 2020, chapter 15). This data format contains all essential information and is open, so it can be further processed without any proprietary restrictions.

The point-light display (PLD) stimuli were rendered using Blender (Blender Community, 2018), an open-source 3D rendering engine that allows flexible creation and editing of scenes, including positioning and configuration of camera viewpoints, and recording of sequential data into various formats (including video). Specifically, we developed a Python plugin that allows Blender to process the MVNX sequences and convert them into a Blender-friendly format, based on hierarchical relationships between different movements in a human skeleton (a process called “rigging”). We have released our MVNX rigging plugin to GitHub under GPLv3 license (see data availability statement, Section 11).

We also developed custom software to assist with the process of blurring faces, in the form of a series of Python scripts that make use of third-party, open-source deep learning models to first detect the dancer's head (Wu et al., 2019), and then identify the pixels that correspond to the face (more details are provided in the following sections). We have also released these scripts to GitHub under GPLv3 license, with the hope that they can be useful to the community (see data availability statement, Section 11).

Finally, the software *Adobe After Effects* 2019 and *Adobe Premiere Pro* 2019 were used for rendering the video clips in postproduction. The online survey tool Limesurvey® was used for the observer experiment, and the experiment was launched via the Prolific® online platform.

### Overall procedure

The recording of the pilot stimuli was carried out by a team of five researchers and three filmmakers. The recording procedure followed the recommended standard practice by the XSENS Company (see the XSENS Manual, 2020); chapters 7 and 8), including body measurements and a calibration routine, before the start of each recording session. At the end of each calibration, the dancer was instructed which sequence to perform and which emotion to express, based on an a priori established list of choreographies and emotion orders. The dancer proceeded to say the name of the sequence and the emotion out loudly. Then, clapped twice to signal the beginning of the sequence and to secure the alignment of XSENS®, video and audio recordings. As specified in more detail in section "Stimuli (of the pilot dataset)", 12 separate choreographies were created for the EMOKINE pilot dataset (i.e., 12 different sequences of movements). Each of these 12 choreographies was then performed by the dancer six times, to express a different emotion at each repetition. Hence, each emotion was expressed once for each of the 12 choreographies. The order of the ‘emotion takes’ for each sequence was always: neutrality, joy, contentment, sadness, fear, and anger. If the dancer was not satisfied with the performance (e.g., made a mistake in the choreography, or felt that the emotion was not expressed), a second (or third) ‘emotion take’ was performed of the same movement until the dancer agreed with the performance. If the dancer made a mistake in the choreography, the stimulus was discarded without further analysis. If the dancer felt the emotion was not expressed, the “best” sequence of these duplicate takes was chosen by the dancer.

Subsequently, the recordings were rendered in the four different visual presentations. This coarse parameterization was devised in order to enable research into how emotion recognition and beauty ratings are affected by these four different levels of information detail in the representation of emotional kinematics.

#### Procedure for human observer experiment

Four separate experiments were set up to allow the 216 stimuli to be rated by four separate groups of participants. Stimuli were divided randomly, but equally between the experiments, to include the same number of stimuli of each type of visual presentation. To ensure that ratings would be equivalent across all four experiments, one sequence (i.e., 6 stimuli × 4 visual presentations = 24 stimuli) was selected and presented in all four experiments, for an interrater reliability check. For the order of stimuli presentation during the experiment, visual presentation was blocked, but stimuli and blocks were randomized across participants.

**Fig. 2:**
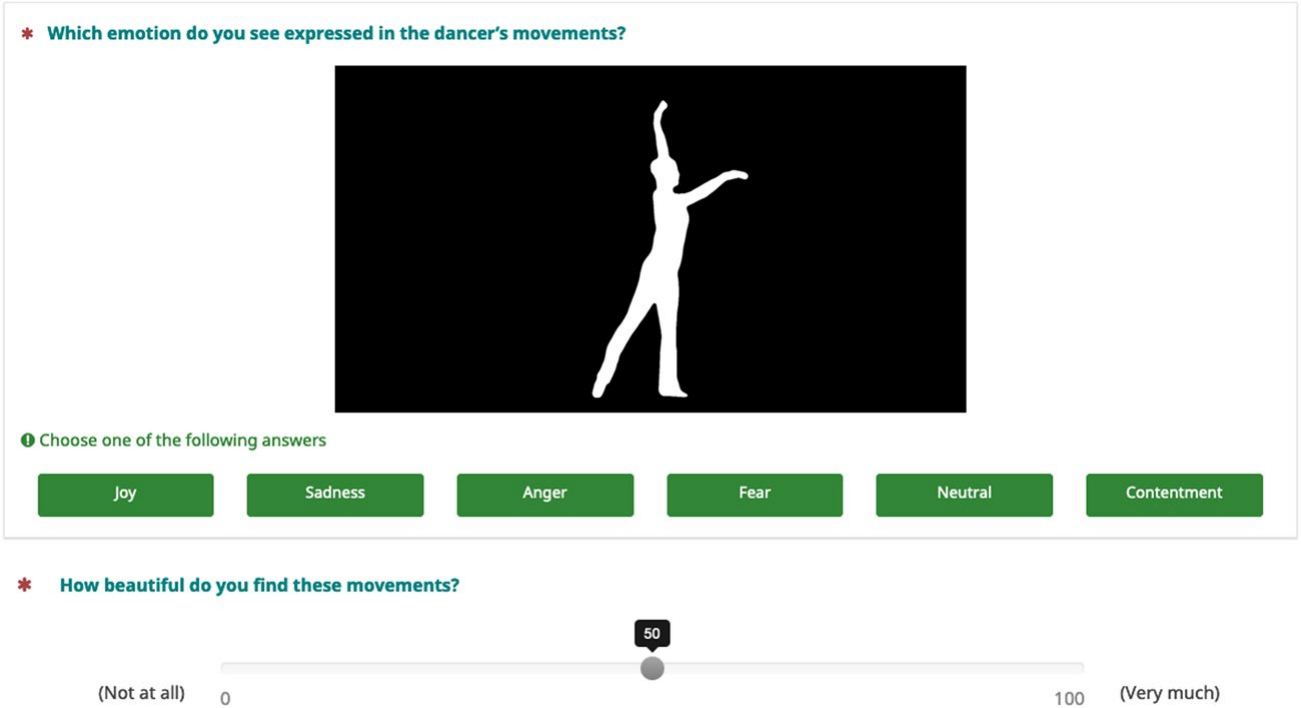
One trial of emotion recognition experiment with human observers. *Note*: Sample trial from the human observer experiment. Stimuli were shown one by one (here, a silhouette video). Participants were instructed to guess the emotion the dancer was expressing (forced choice task; anger, contentment, fear, joy, neutrality, sadness), and to rate how beautiful they found the movement (slider question; 0 = not beautiful; 100 = very beautiful)

Participants watched the stimuli one by one, and rated, first, what emotion they recognized in the movement (forced choice task; anger, contentment, fear, joy, neutrality, sadness), and then, how beautiful they found the movement (Likert scale; 0 = not beautiful; 100 = very beautiful). Participants could only watch each stimulus once and were then asked to provide their rating. As this was an online experiment operated via Prolific®, viewing angle and distance were not controlled. However, filters on Prolific were set so that the experiment could only be performed on a computer desktop (not on a tablet or mobile phone). Figure 2 sets out one trial of the observer experiment.

After the four blocks (with breaks in between), participants were asked to fill in three questionnaires: the Aesthetic Responsiveness Assessment (AReA) (Schlotz et al., 2020), the Interpersonal Reactivity Index (IRI) (Davis, 1980), and demographics questions. The questionnaire data are not presented here.

## Pilot dataset specifications

### Stimuli (of the pilot dataset)

**Fig. 3:**
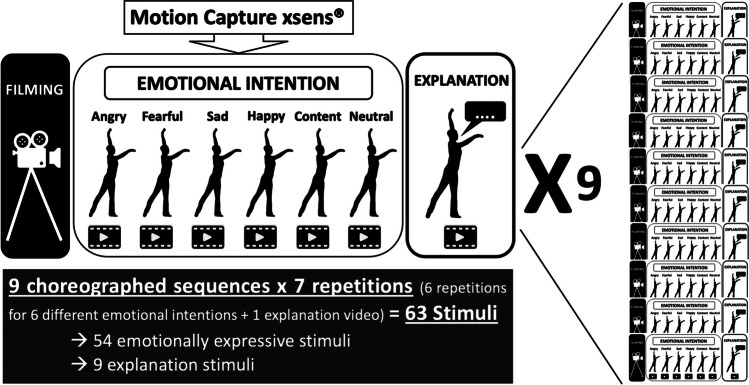
Pilot dataset creation. *Note*: The dancer wore an XSENS® Motion Capture Suit LINK while performing nine different sequences of choreographed movements (see Table 2). She repeated these choreographies seven times each. Six times she performed the movements with different emotional intentions at each repetition (anger, contentment, fear, joy, neutrality, sadness). The seventh time she explained the movements, as for a dance class instruction. The latter stimuli were included in a different experiment (Schmidt et al., 2023), but are also included in the present dataset for sake of completeness

Originally, 12 dance sequences were created by the dancer. However, three of these were deemed not good enough by the dancer and therefore discarded before any further analysis, yielding a total of nine sequences included in the subsequent computations presented here. Each sequence was performed six times to express a different emotional intention at each repetition, namely, anger, contentment, fear, joy, neutrality, sadness; i.e., 9 sequences × 6 emotions = 54 emotional dance movement stimuli. In addition, for each sequence, the dancer did a seventh repetition of the sequence, during which she explained the movements while doing them, like an instruction video for a dance class; yielding nine explanation videos (used elsewhere; Schmidt et al., 2023), yet the videos are provided here as part of the full EMOKINE dataset). Therefore, the total number of stimuli in the EMOKINE dataset is 63 (see Fig. 3).

**Table 2:** Movement sequences of the nine EMOKINE pilot dataset choreographies

Sequence no.	Sequence movements	Repetitions
Seq1	Arms: bras-bas position move up to both arms in 2nd position, palms down, elbows lead. Then, back to bras-bas. Port-de-bras to 1st position, and back to bras-bas. Legs: remain feet parallel, first position.	seq1_angry seq1_content seq1_fearful seq1_joy seq1_neutral seq1_sad seq1_explanation
Seq2	Arms: Move from bras-bas via port-de-bras to first position, open to second, then allongé arms slightly over 180 degrees, palms turn downward, then back to bras-bas position. Legs: remain feet parallel, first position.	seq2_angry seq2_content seq2_fearful seq2_joy seq2_neutral seq2_sad seq2_explanation
Seq3	Arms: bras-bas position move up to both arms in 2nd position, palms down, elbows lead. Then rotate to open 4th allongé, leading with the left arm front, right arm back, then rotate the right arm front, left back. Legs: remain feet parallel, first position.	seq3_angry seq3_content seq3_fearful seq3_joy seq3_neutral seq3_sad seq3_explanation
Seq4	Arms: from bras-bas position move right arm though 1st position, up to 5th. Then left arm follows the same path. Both arms open simultaneously to 2nd position (held elbows in 2nd), subtle allongé and close arms in bras-bas. Make a final allongé movement from bras-bas to 5th position, palms turned out, elbows supple (“swan arms”). Legs: remain feet parallel, first position.	seq4_angry seq4_content seq4_fearful seq4_joy seq4_neutral seq4_sad seq4_explanation
Seq5	Arms: from bras-bas position move right arm though 1st position, up to 5th. Then left arm follows the same path. Both arms lower back down simultaneously to 1st position, then open in second position, allongé, close in bras-bas. Legs: remain feet parallel, first position.	seq5_angry seq5_content seq5_fearful seq5_joy seq5_neutral seq5_sad seq5_explanation
Seq6	Arms: from bras-bas position move both arms to 1st position (elbows held), then allongé arms to open 4th position croisé to the right, pass through 1st position, then to open 4th position croisé to the left. Back to 1st position and move arms back down to bras-bas with elbows held. Legs: remain feet parallel, first position.	seq6_angry seq6_content seq6_fearful seq6_joy seq6_neutral seq6_sad seq6_explanation
Seq7	From parallel 0-position, arms bras-bas: arms and legs together: Slide through plié to the right, to an open wide second position (legs), while arms go from bras-bas to 1st position. Rotate to arms croisé open allongé 4th position, legs, right leg tendu derriere. Pass back through 2nd position, arms 1st. Rotate to left, again, arms croisé open allongé 4th position, legs, right leg tendu derriere. Back to 2nd position legs, plié, arms open to second position, stretch arms allongé as legs stretch second position. Close in from second position turned out, right leg slides back in, to parallel 0-position.	seq7_angry seq7_content seq7_fearful seq7_joy seq7_neutral seq7_sad seq7_explanation
Seq8	From parallel 0-position, arms bras-bas: arms and legs together: step with the left leg to an open efface 4th position with the legs, while arms go up, through 1st position to 5th position. Open arms to 2nd position, allongé arms to an open third (left arm back, right arm croisé front), then pass back through 2nd position with the arms and make an open third again, to the other side (right arm back, left arm effacé front). Arms back to second position. Then, at the same time, close arms to bras-bas and step left leg back to parallel 0-position.	seq8_angry seq8_content seq8_fearful seq8_joy seq8_neutral seq8_sad seq8_explanation
Seq9	Arms and legs together: step out with left foot to an open second, left arm makes a sweeping movement, port-de-bras through 1st position to second, but upper body tilted, so left hand almost touches floor. Again, simultaneously, left arm closes up to 5th position, and does cambré to the right while left leg stretches, so a diagonal line between left toe and left arm is formed. Right leg is 2nd position plié, head looks to the left. Left arm opens to 2nd position, while legs plié in 2nd position. Then arm makes a fast port-de-bras through bras-bas then 1st and up to 5th, while legs do a balancé (a round shape is created in the air). During the movement, the right arm and hand are fairly still, right hand resting lightly on the lower right hip. When left arm reaches second position again, freeze and legs close back to 0-position and arms back to bras-bas.	seq9_angry seq9_content seq9_fearful seq9_joy seq9_neutral seq9_sad seq9_explanation

Choreographies of the nine movement sequences included in the EMOKINE dataset. The movements contained in each sequence are set out in this table using Western classical ballet vocabulary

Seven of the nine sequences that had been choreographed involved only arm movements, while the lower body was held relatively still (only one step to the side). The two remaining sequences involved some side steps (“second-position” in the ballet syllabus). However, as a first step towards quantifying the kinematics of emotional expressivity in dance, the dancer kept most of the emotional expressivity in the EMOKINE dataset to the arms, following previous dataset creations (that did not use motion capture), which has focused on the arms only (Sawada et al., 2003) (see Table 2 for an overview of the choreographies of each sequence’s movements).

**Fig. 4:**
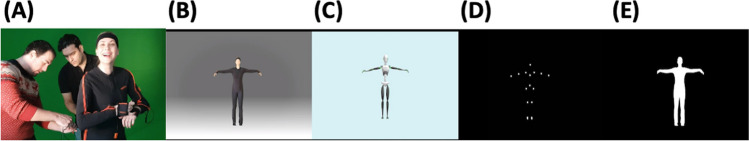
The four visual presentations of the EMOKINE pilot dataset. *Note*. **(A)** One dancer performed nine dance sequences of eight counts six times with six different emotional intentions, while wearing an XSENS® Motion Capture Suit in front of a greenscreen. Filming was done with a Panasonic camera. **(B)** Full-light display (FLD) dancer with blurred face: Background grey set with Adobe After Effects Keylight effect. Face blurred with automated computational methods (Cheng et al., 2019; Nirkin et al., 2017). **(C)** XSENS® Avatar dancer: Extracted from XSENS® system propriety software (XSENS Manual, 2020). **(D)** Point-light display (PLD) dancer: XSENS® output data with skeleton information (MNVX) fed into a customized Blender module and white spheres attached to skeleton (Blender Community, 2018; Schepers & Giuberti, 2018). **(E)** Silhouette dancer: Adobe After Effects Keylight effect used to remove background (greenscreen). Adobe After Effects Opacity function used to color foreground (dancer) white

In post-production, all 63 pilot stimuli (the 54 emotional stimuli plus the nine explanation videos) were rendered in four different visual presentations (avatars, full-light displays (FLDs), point-light displays (PLDs), silhouettes). None of the stimuli contains facial information, there is no costume, color, or music in the clips. Each clip was faded in and out and contains one full dance phrase (eight counts in dance theory) (see Fig. 4 for the four visual presentations).

The EMOKINE pilot dataset is available for download online, and a selection of stimuli has been used in published work (Schmidt et al., 2023). However, please note that this dataset was created to enable the development of the EMOKINE software and contains portrayals from only one dancer. To ensure generalizability of results from future research using the EMOKINE software, new datasets should be created that include portrayals from more than one dancer. We hope that the details about the stimuli creation procedure set out above may help this endeavor.

#### Full-light displays (FLDs) with blurred face

For the full-light displays (FLDs) with blurred face, videos were rendered by importing them into Adobe Premiere Pro. The videos were trimmed to the start and end points of the movements with the help of a dancer (academic dance sequences have specific start and end points that are only detectable for the expert). Each clip was rendered into a separate file in an uncompressed format and the title was added, as specified verbally by the dancer during the recording. In this saving procedure, the sound track (ambient noise) of the clips was removed. Then, all rendered files were imported to Adobe After Effects. The “Keylight” effect was used to set the background to be a shade of grey.

Blurring the face required locating the pixels that correspond to the face, which can be a very time-consuming task if done manually for video datasets. To speed up the process, we developed rigging software for a semi-automated pipeline. Each video was split in consecutive, deinterlaced image frames that were processed separately. For each image, the Detectron2 deep learning model for human keypoint estimation was used (Wu et al., 2019). Since we only had a single person on static background, averaging any detected keypoints for the nose, eyes and ears provided a very robust estimation of the head position, and given that the dancer was always more or less centered and at the same distance from the camera, a fixed-length frame of 140 × 140 pixels around the estimated head position was used, in order to extract a patch containing the head. This allowed the face segmentation model by Nirkin and colleagues (2017) to produce a binary mask that accurately matched the actual face at pixel level. This binary mask was then translated from the head-patch back to the main image. The resulting masks for the whole dataset were then grouped and paired with the corresponding videos in order to blur the faces at the regions where the masks were active.

#### Silhouettes

To render the footage into silhouette dancer videos, all footage was imported into Adobe Premiere Pro as before. Here, the “Keylight” effect was added, and settings adapted to remove the background from each clip, and the “Level” effect (setting: output black = 255) was added to each clip to color the extracted foregrounds white (the visible dancer silhouette). “Opacity” keyframes were then added to the beginning and the end of each clip to allow for a fade-in and fade-out of each clip (eight frames). Finally, each clip was rendered as a separate file in H264 format (see Fig. 2).

#### Point-light displays (PLDs)

The point-light display (PLD) videos were created using the XSENS output data, MNVX (Blender Community, 2018; XSENS Manual, 2020). The MNVX file contains information about the skeleton (bone geometry and connections), and each “frame” (240 frames per second) contains kinematic information about the position and angle of the bones. We wrote a custom Blender plugin (Blender Community, 2018) that read each MVNX file and created a skeleton with the corresponding geometry and connections. Then, the module read the frame information and created an animation. Based on previous models for marker placement, namely the frontal view of the Plug-In Gait Model (Kainz et al., 2017; Piwek et al., 2016), we identified a series of key landmarks on the skeleton, and attached a white sphere to each landmark, in order to create the “light points” that convey the information about the movements. For the video rendering, we positioned a virtual camera “in front of” the PLDs with an angle, position and focal length that closely resembled the data obtained with the video camera (for an example of the result, see Fig. 3). Then, the skeleton was made transparent (making it black on a black background) and the spheres bright white (increasing the contract), allowing to extract the pixel-position of each point in the rendering. Videos were faded in and out.

#### XSENS avatar dancer

The XSENS® avatar dancers were extracted from the propriety software of the XSENS® system (XSENS Manual, 2020).

### Data formats (of the pilot dataset)

Beyond the already-discussed four modalities for the stimuli, we include with the EMOKINE pilot dataset, several modalities of data records that we recommend to use in the future, as they may help with downstream tasks. The pilot files are available on Zenodo (see Section 11; Data Availability Statement), and consist of the following file formats MVNX, comma-separated values, and camara position (CamPos). Extensive details are provided in the readme files along with the data and software on Zenodo and GitHub.

#### MVNX files

We include the raw MVNX motion capture recordings, as produced by the XSENS® software for the pilot dataset.

#### Comma-separated values (CSV) files

To facilitate easy integration with other data analysis tools, we recommend converting a subset of the MVNX files into comma-separated value (CSV) files. For each sequence and emotion, we extract per-keypoint time series for position, orientation, velocity, angular velocity, acceleration, angular acceleration, center of mass and foot contacts. In the EMOKINE software package, we provide the script and instructions to perform this conversion.

#### Camera position (CamPos) files

While the positional data in the MVNX files is provided in a global three-dimensional frame of reference, the stimuli are rendered from a specific camera perspective. We use Blender to extract the positions of the bones and PLD spheres relative to the camera, as *x*/*y*/*depth* coordinates, where *x* goes from 0 (leftmost pixel) to 1 (rightmost pixel), *y* from 0 (bottom pixel) to 1 (top pixel) and *depth* is provided in meters. The result, dubbed here CamPos (for camera positions), is provided as JSON files containing the time series in frames at 60 Hz, where each frame contains the camera-relative positions. This can be useful for example in analyzing kinematic features from the perspective of the observer. In the EMOKINE software package, we also provide the script and instructions to produce these files.

#### Using the EMOKINE software with data obtained from other motion-capture systems

Although the EMOKINE pilot dataset was recorded via the XSENS system, most of the EMOKINE software provided here can be directly applied to data obtained from other motion capture systems with little to no modification. Specifically, only the files “1a_mvsn_to_csv.py” and “1b_mvnx_blender.py” are relevant to the MVNX formatted data. These data are then converted to tabular format (see GitHub repository for examples), which is then consumed by the remaining scripts (together with plain video data whenever needed, e.g., for the file “2b_face_blur.py”). Researchers intending to use this software with motion capture data from other systems simply need to ensure that their data follows the same tabular format, and that they have video data available whenever needed (e.g., for face blur or silhouette extraction).

## Kinematic features

Making use of the Silhouette, MVNX, and CamPos data modalities, we compute a series of kinematic features. We extracted 32 statistics from 12 kinematic features. We group the extracted kinematic features in the following categories: Speed and Acceleration (speed, acceleration, angular speed and angular acceleration; Section "Speed and acceleration"), Expansion/Contraction (limb contraction, distance to center of mass; Section "Expansion/contraction"), Movement Activity (quantity of motion, QoM, ratio; Section "Movement activity"), Fluidity/Smoothness (dimensionless jerk (integral); Section "Fluidity/smoothness"), Body Tilt (head angle, with regards to vertical axis and with regards to back; Section "Body tilt"), and Space (convex hull 2d and 3D; Section "Space"). For each of these, we computed the per-sequence *average*, *median absolute deviation* (MAD) and *maximum value* as described above.

The resulting computed features for each sequence and emotion are provided in the EMOKINE dataset, and the script and instructions to compute them from the raw data are included in the EMOKINE software package. In Section "Validation of kinematic features and results of observer experiments", we demonstrate the usefulness of these features and the meaningfulness of the EMOKINE data through a series of quantitative and qualitative analyses.

Before we outline each kinematic feature in detail, we give an overview of the math behind the kinematic features. More formally, for each sequence $s\left(t\right)\in \mathcal{S}$ (from the 63 total in the EMOKINE dataset $\mathcal{S}$), we have $\{{K}_{i}^{\left(s\right)}\left(t\right){\}}_{i=1}^{12}$ nonnegative scalar features, where $t\in \{0,\dots ,{T}_{s}\}$ indicates discrete time with a duration of ${T}_{s}$ frames. Thus, ${K}_{i}^{\left(s\right)}\left(t\right):\hspace{0.17em}\mathcal{S}\mapsto {\mathbb{R}}_{>0}^{{T}_{s}}$ is the kinematic feature of sequence $s$ out of a total of 12 kinematic features, represented by a nonnegative vector of dimension ${T}_{s}$.

Some of the kinematic features were extracted directly from the MVNX files as provided by the XSENS software (see Section "Pilot dataset specifications" for more details), while others were extracted from the CamPos data (see Section 3.3.3.) and the silhouette stimuli videos (see Section "Silhouettes"). In the following sections, we describe in detail how kinematic features were extracted and/or computed. This information is summarized in Tables 3 and 4.

**Table 3:** Formulas for kinematic feature extraction

Name	Source	Formula	Unit
Speed	MVNX	${{\text{v}}}_{{\text{j}}}\left({\text{t}}\right):=\parallel {\nabla }_{{\text{t}}}{{\text{p}}}_{{\text{j}}}\left({\text{t}}\right){\parallel }_{2}$	$\frac{m}{s}$
Acceleration	MVNX	${{\text{a}}}_{{\text{j}}}\left({\text{t}}\right):=\parallel {\nabla }_{{\text{t}}}^{2}{{\text{p}}}_{{\text{j}}}\left({\text{t}}\right){\parallel }_{2}$	$\frac{m}{{s}^{2}}$
Angular speed	MVNX	$\dot{{\upomega }_{\upxi }}\left({\text{t}}\right):=\parallel {\nabla }_{{\text{t}}}{\upomega }_{{\text{j}}}\left({\text{t}}\right){\parallel }_{2}$	$\frac{rad}{s}$
Angular acceleration	MVNX	$\ddot{{\upomega }_{\upxi }}\left({\text{t}}\right):=\parallel {\nabla }_{{\text{t}}}^{2}{\upomega }_{{\text{j}}}\left({\text{t}}\right){\parallel }_{2}$	$\frac{rad}{{s}^{2}}$
Limb contraction	MVNX	${{\text{l}}}_{{\text{c}}}\left({\text{t}}\right):=\frac{1}{4}\left(\parallel {{\text{p}}}_{{\text{h}}}\left({\text{t}}\right)-{{\text{p}}}_{{\text{a}}}\left({\text{t}}\right){\parallel }_{2}+\cdots \right)$	$m$
CoM distance	MVNX	${m}_{j}\left(t\right):=\parallel {p}_{j}\left(t\right)-\upmu \left(t\right){\parallel }_{2}$	$m$
Quantity of motion	Silhouette	$q\left(t\right):=\frac{{\left|{Q}_{\updelta }\left(t\right)\right|}_{1}}{{\left|f\left(t\right)\right|}_{1}}$	$\mathrm{\varnothing }$
Dimensionless jerk	MVNX	${\lambda }_{j}\left(t\right):=\frac{{\left({T}_{s}\right)}^{5}}{{\Delta }_{p}^{2}}{\int }_{t=0}^{{T}_{s}}\dot{{a}_{j}}{\left(t\right)}^{2}$	$\mathrm{\varnothing }$
Head angle wrt. back	MVNX	$\mathrm{\alpha }\left(t\right):=co{s}^{-1}\left({u}_{ab}{\left(t\right)}^{\mathrm{\top }}{u}_{bc}\left(t\right)\right)$	$rad$
Head tilt wrt. vertical	MVNX	$\upbeta \left(t\right):={\text{co}}{s}^{-1}\left({u}_{\uparrow }{\left(t\right)}^{\mathrm{\top }}{u}_{bc}\left(t\right)\right)$	$rad$
Convex hull 3D	MVNX	$volume\left({\mathcal{C}}_{3\mathcal{D}}\left(t\right)\right)$	${m}^{3}$
Convex hull 2D	CamPos	$area\left({\mathcal{C}}_{2\mathcal{D}}\left(t\right)\right)$	$\mathrm{\varnothing }$

Summary of the computations used to extract the kinematic features. Some were extracted directly from the MVNX files, as provided by the XSENS® software (see Section 3.3. for more details), while others were extracted from the CamPos data (see Section 3.3.3.) and the silhouette stimuli videos (see Section "Silhouettes"). We refer the reader to Sections "Speed and acceleration" for the comprehensive definitions of the quantities

We aggregate each kinematic feature across time $t$ to obtain a single scalar statistic that summarizes the kinematics of each sequence $s$. The following aggregation techniques are used in multiple features:

- Average: $${\overline{K} }_{i}^{\left(s\right)}:=\frac{1}{{T}_{s}}{\sum }_{t=1}^{{T}_{s}}{K}_{i}^{\left(s\right)}\left(t\right)$$
- Median absolute deviation (MAD): ${\widetilde{K}}_{i}^{\left(s\right)}:=\underset{t}{{\text{median}}}\left(\underset{t}{| \mathit{median}}\left({K}_{i}^{\left(s\right)}\left(t\right)\right)-{K}_{i}^{\left(s\right)}\left(t\right)|\right)$
- Maximum:$${\widehat{K}}_{i}^{\left(s\right)}:=\underset{t}{{\text{max}}}\left({K}_{i}^{\left(s\right)}\left(t\right)\right)$$

Qualitatively, the ${K}_{i}^{\left(s\right)}\left(t\right)$ features tell us “how much” of a given feature is given at each timepoint. Then, the main difference between these three aggregations is their sensitivity to outliers: ${\widehat{K}}_{i}^{\left(s\right)}$ is the most sensitive, and ${\widetilde{K}}_{i}^{\left(s\right)}$ is the least sensitive. The average: ${\overline{K} }_{i}^{\left(s\right)}$ lies inbetween. Varying sensitivity to outliers is important if a sequence relies on punctual strong phenomena to convey crucial information (e.g., a short burst in velocity in a generally slow sequence will still have a large maximum), or conversely, to recover the underlying information in cases, where the sequence is exposed to outliers (e.g., if a movement is supposed to be smooth, but is slightly shaky, or contaminated with noise).

### Speed and acceleration

Speed is one of the most frequently explored kinematic parameters, and research suggests that it plays a substantial role in an observer’s ability to distinguish between specific kinds of emotional expressivity. The majority of this work suggests that slow movements are associated with sadness, and in some cases with expressions of neutrality and fear, while fast movements are typically associated with happiness (or joy) and anger (Bernhardt & Robinson, 2007; Crane & Gross, 2007; Crane & Gross, 2013; Gross et al., 2010; Halovic & Kroos, 2018; Masuda et al., 2010; Montepare et al., 1999; Roether et al., 2009; Smith & Pollick, 2022).

Acceleration is less studied in relation to observer judgements of emotional expressivity in the kinematics literature. But in a study conducted by Sawada and colleagues, a similar pattern emerged across these movement features. Namely, they found that high acceleration in arm movements was associated with anger, and low acceleration was associated with sadness (Sawada et al., 2003). We here provide a series of speed and acceleration related features for to enrich emotional kinematics research in the future; speed (Section "Speed"), acceleration (Section "Acceleration"), angular speed (Section "Angular speed") and angular acceleration (Section "Angular acceleration").

#### Speed

Velocity is a vector provided by the MVNX system that points in a specific 3D direction, and speed is the “length” of the vector. This length tells us how fast is a given keypoint moving in that direction, in meters per second. More formally, if the position in meters of a given joint $j$ in the 3D space at time $t$ is:

$${p}_{j}\left(t\right)=\left(\begin{array}{c}x\\ y\\ z\end{array}\right)$$

Where $x$ is aligned (and pointing to) the magnetic north, $y$ is aligned (and pointing to) the west, and $z$ is pointing up (for more details, see the XSENS Manual (2020; section 23.8). Then, the velocity $v$ is the derivative of the position with respect to time (${\nabla }_{t}$), and our *speed* feature is the Euclidean norm of the velocity, i.e. ${\Vert {{\text{v}}}_{{\text{j}}}\left({\text{t}}\right)\Vert }_{2}$:

$${{\text{v}}}_{{\text{j}}}\left({\text{t}}\right)={\nabla }_{{\text{t}}}{{\text{p}}}_{{\text{j}}}\left({\text{t}}\right)=\left(\begin{array}{c}{\nabla }_{{\text{t}}}{\text{x}}\\ {\nabla }_{{\text{t}}}{\text{y}}\\ {\nabla }_{{\text{t}}}{\text{z}}\end{array}\right)$$

Note that, in discrete time, this quantity could be approximated by computing $\frac{p\left(t+{\Delta }_{{\text{t}}}\right)-p\left(t\right)}{{\Delta }_{t}}$, where ${\Delta }_{{\text{t}}}$ is a small amount of discrete time (e.g., one frame), but in this case it is not necessary since it is provided directly by the MVNX file, and estimated by the XSENS system using a proprietary algorithm; see XSENS Manual (2020; section 23.8). We provide the average, MAD and maximum velocity for each joint $j$ and sequence $s$ in EMOKINE.

#### Acceleration

For each joint and timepoint we define the acceleration ${a}_{j}\left(t\right)$ as a three-dimensional vector, encoding the rate of change in the speed with respect to time. Our *acceleration* feature is then the Euclidean norm of that vector i.e., ${\Vert {a}_{j}\left(t\right)\Vert }_{2}$ with:

$${a}_{j}\left(t\right)={\nabla }_{t}^{2}{p}_{j}\left(t\right)={\nabla }_{t}{v}_{j}\left(t\right)=\left(\begin{array}{c}{\nabla }_{t}^{2}x\\ {\nabla }_{t}^{2}y\\ {\nabla }_{t}^{2}z\end{array}\right)$$

The acceleration vectors ${a}_{j}\left(t\right)$ are also estimated through the XSENS proprietary algorithm (XSENS Manual, 2020; section 23.8) and provided directly through the MVNX files. The acceleration is conceptually associated to the “force” applied to a joint. As with the *speed* feature, the Euclidean norm does not convey information about the directionality. We provide the average, MAD and maximum acceleration for each joint $j$ and sequence $s$ in EMOKINE.

#### Angular speed

Joints not only have positions, but they also have orientations. A joint can change position without changing orientation (e.g., walking with a straight head), and vice versa (rotating the neck while standing still). The angular speed focuses on the orientation: It measures the change of “angle” as a function of time, so instead of meters per second, we have radians per second. If a dancer is rotating a full circle per second, then the angular speed of their body would be $2\pi$ radians per second ($2\pi$ radians = 360 degrees).

In the XSENS system, each keypoint is considered the beginning of a segment (can be thought of as a "bone") with its own, local three-dimensional coordinate system. When the subject stands in T-pose, all local coordinate systems are aligned with the global system (see Fig. 92 in section 23.5 of the XSENS Manual,  2020). Then, the segment rotations follow the *Z* (flexion/extension), *X* (abduction/adduction), *Y* (internal/external) convention (see XSENS Manual, 2020; section 23.6, for exhaustive details). More formally, for each segment $\upxi$, the orientation in radians ${\upomega }_{\upxi }\left({\text{t}}\right)$ is given as a three-dimensional vector in Euler representation, which varies as a function of time:

$${\upomega }_{\upxi }\left({\text{t}}\right)=\left(\begin{array}{c}{\upomega }_{{\text{x}}}\\ {\upomega }_{{\text{y}}}\\ {\upomega }_{{\text{z}}}\end{array}\right)$$

Then, the *angular speed* feature is the Euclidean norm of the derivative of ${\omega }_{\xi }\left(t\right)$ with respect to time, i.e., $\dot{{{\text{w}}}_{\upxi }}\left({\text{t}}\right)={\Vert {\nabla }_{{\text{t}}}{\upomega }_{\upxi }\left({\text{t}}\right)\Vert }_{2}$, given in $\frac{rad}{s}$. Like the rest of quantities presented so far, this quantity is estimated by the XSENS proprietary algorithm, and directly provided via the MVNX file. Analogously to the linear velocity previously discussed, the Euclidean norm retains the information about the amount of angular speed, but does not contain information about the specific directions. We provide the average, MAD and maximum angular velocity for each joint $j$ and sequence $s$ in EMOKINE.

#### Angular acceleration

Analogously to the case of linear acceleration, angular acceleration is the second derivative of angle with respect to time, i.e., $\ddot{{w}_{\upxi }}\left(t\right)={\Vert {{\nabla }^{2}}_{t}{\omega }_{\xi }\left(t\right)\Vert }_{2}$. Like the rest of quantities presented so far, this quantity is estimated by the XSENS proprietary algorithm, and directly provided via the MVNX file. The Euclidean norm retains the information about the amount of angular acceleration but does not contain information about the specific directions. We provide the average, MAD, and maximum angular velocity for each joint $j$ and sequence $s$ in EMOKINE.

### Expansion/contraction

Body expansion and contraction is another kinematic feature of movement commonly explored in emotion perception research. However, unlike with speed, the results in this area do not present such a clear pattern of associations, likely because many datasets used in this area focus on emotional actions (instead of the same-sequence approach proposed in EMOKINE).

Of the available literature, most research in this area seems to agree that expansion is associated with happiness or joy, and some suggest that it also leads to the perception of anger (Camurri et al., 2003; Gross et al., 2010; Gross et al., 2012; Masuda et al., 2010; Montepare et al., 1999; Shafir, 2016; Shikanai et al., 2013; Wallbott, 1998). Castellano and colleagues’ study is a notable exception in that they found anger to be associated with contraction instead (Castellano et al., 2007). However, contraction is more commonly noted to align with the perception of fear and sadness (Camurri et al., 2003; Masuda et al., 2010; Shafir et al., 2016; Shikanai et al., 2013; Wallbott, 1998), and in some cases with neutral expressivity (Montepare et al., 1999). For body expansion/contraction, the EMOKINE framework includes limb contraction (Section "Limb contraction") and distance to center of mass (Section "Distance to center of mass (CoM)").

#### Limb contraction

Limb contraction regards the positions of five keypoints: head, right hand, left hand, right toe and left toe (Poyo Solanas et al., 2020). Respectively: $\left({p}_{h}\left(t\right),{p}_{a}\left(t\right),{p}_{b}\left(t\right),{p}_{c}\left(t\right),{p}_{d}\left(t\right)\right)$. Then, at each timepoint , this metric consists in the mean Euclidean distance between each of the four extremity endpoints and the head, i.e.,

$${{\text{l}}}_{{\text{c}}}\left({\text{t}}\right)=\frac{1}{4}\left({\Vert {p}_{h}\left(t\right)-{p}_{a}\left(t\right)\Vert }_{2}+{\Vert {p}_{h}\left(t\right)-{p}_{b}\left(t\right)\Vert }_{2}+{\Vert {p}_{h}\left(t\right)-{p}_{c}\left(t\right)\Vert }_{2}+{\Vert {p}_{h}\left(t\right)-{p}_{d}\left(t\right)\Vert }_{2}\right)$$

This metric is a proxy for body contraction, with the idea that contracted bodies tend to have shorter distances between the limb endpoints and the head, while expanded poses tend to have longer distances. We provide the average and MAD limb contraction for each joint $j$ and sequence $s$ in EMOKINE.

#### Distance to center of mass (CoM)

At each timepoint $t$, and together with the joint positions ${p}_{j}\left(t\right)$, the XSENS system also estimates and retrieves the position of the person's *center of mass* $\upmu \left(t\right)$, in meters, which is a “weighted average” among all the points in the body, thus representing the idea of its “central point”. For each sequence in EMOKINE and for each keypoint $j$, we compute the CoM distance between each keypoint position ${p}_{j}\left(t\right)$ and the CoM as follows:

$${m}_{j}\left(t\right)=\parallel {p}_{j}\left(t\right)-\mu \left(t\right){\parallel }_{2}$$

We then compute and retrieve the average and MAD.

### Movement activity

Movement activity has been examined in a number of ways in the kinematics literature depending on the particular movement stimuli used. For example, in studies exploring the kinematics of walking motions it is typically measured via step frequency (e.g., Crane & Gross, 2007). More often, however, it is quantity of motion that is assessed. Research in this area, as with speed, suggests that high movement activity is typically associated with the portrayal of joy and anger and low movement activity is associated with sadness, fear, and sometimes neutral expressivity (Bernhardt & Robinson, 2007; Camurri et al., 2003; Crane & Gross, 2007; Crane & Gross, 2013; Gross et al., 2010; Halovic & Kroos, 2018; Masuda et al., 2010; Montepare et al., 1999; Roether et al., 2009; Shikanai et al., 2013). Wallbott (1998) is an exception to this, in that they found variations in movement activity to distinguish between happiness with different levels of intensity; high activity was associated with “elated joy”, but the more general “happiness” was associated with low movement activity. Based on this research, the EMOKINE framework includes Quantity of Motion (QoM) as a measure of movement activity (Section "Quantity of motion (QoM)").

#### Quantity of motion (QoM)

Unlike the other quantities presented so far, the QoM is not extracted from the MVNX sequential data. Instead, the input are silhouette videos at 25 fps, where each frame is a Boolean matrix ${\text{f}}\left({\text{t}}\right)\in \{\mathrm{0,1}{\}}^{1080\times 1920}$, with pixel values of 0 corresponding to the background, and values of 1 to the dancer. The QoM is a time-dependent feature that can be intuitively understood as a ratio between how much has the silhouette moved in the recent past, in proportion to how big the silhouette is right now (Castellano et al., 2007). More formally, given a time span of $\updelta$ frames, we make use of the boolean operations of pixel-wise union ($\vee$), intersection ($\wedge$), negation ($\neg$) and sum (${|\cdot |}_{1}$) to define the QoM $q\left(t\right)$ as follows:

$${Q}_{\updelta }\left(t\right)=\left({\bigvee }_{i=1}^{\updelta }f\left(t-i\right)\right)\wedge \neg f\left(t\right)$$

$$q\left(t\right)=\frac{{\left|{Q}_{\updelta }\left(t\right)\right|}_{1}}{{\left|f\left(t\right)\right|}_{1}}$$

The Boolean array ${Q}_{\updelta }\left(t\right)\in \{\mathrm{0,1}{\}}^{1080\times 1920}$, also called the *silhouette motion mask*, is active for the pixels that were active immediately before the current time and are not currently active. Thus, it encodes the “recent activity”: it is all zeros if there is no movement, and it contains more active pixels as movement increases.

Then, the QoM is the ratio between the sum of active pixels in ${Q}_{\updelta }\left(t\right)$ and the sum of currently active pixels. Note that the QoM is a full-body quantity and does not depend on a given joint. Therefore, for each sequence, we provide only one scalar average, MAD, and integral QoM. While the average QoM is trivially the integral divided by the number of frames, we included both here for convenience, since the integral depends on the sequence length and is a quantity of interest in the literature.

### Fluidity/smoothness

Comparatively, there is less research examining the role of movement fluidity in the perception of emotional expressivity, but there does exist some evidence to suggest that movement fluidity is associated with happiness or joy, while stiff or low-fluidity motion is associated with anger (Montepare et al., 1999) and other negative valence emotions like grief and fear (Camurri et al., 2003). For the EMOKINE framework, we computed dimensionless jerk (integral), as a measure of movement fluidity/smoothness (Section "Dimensionless jerk (integral)").

#### Dimensionless jerk (integral)

Based on Hogan & Sternad (2009), this feature proposes a variation of the jerk, which is the time-derivative of acceleration (Hogan & Sternad, 2009):

$$\dot{{a}_{j}}\left(t\right)={\nabla }_{t}{a}_{j}\left(t\right)={\nabla }_{t}^{3}{p}_{j}\left(t\right)=\left(\begin{array}{c}{\nabla }_{t}^{3}x\\ {\nabla }_{t}^{3}y\\ {\nabla }_{t}^{3}z\end{array}\right)$$

In order to quantify smoothness in trajectories from ${t}_{1}$ to ${t}_{2}$ the integral of the square jerk is typically used in the literature, i.e., ${\int }_{{t}_{1}}^{{t}_{2}}\dot{{a}_{j}}{\left(t\right)}^{2}$. One major problem with this, as pointed out by Hogan & Sternad (2009), is that this yields a high-order polynomial unit of $\frac{lengt{h}^{2}}{tim{e}^{5}}$ being sensitive to noise and changes in scale. They propose a dimensionless variant, where the integral is multiplied by $\frac{{\left({t}_{2}-{t}_{1}\right)}^{5}}{{\Delta }_{p}^{2}}$ , where ${{\varvec{\Delta}}}_{{\varvec{p}}}$ is the extent of the length achieved between ${t}_{1}$ and ${t}_{2}$ (i.e., if in a sequence the individual moves more, ${{\varvec{\Delta}}}_{{\varvec{p}}}$ will be larger for that sequence). The result yields a notion of movement smoothness that is normalized against duration and size, and (by design) it is also void of any units, hence the dimensionless characterization. For each sequence *s*, we provide the dimensionless jerk between ${t}_{1}=0$ and ${t}_{2}={T}_{s}$, i.e.:

$${\uplambda }_{j}\left(t\right)=\frac{{\left({T}_{s}\right)}^{5}}{{\Delta }_{p}^{2}}{\int }_{t=0}^{{T}_{s}}\dot{{a}_{j}}{\left(t\right)}^{2}$$

Note that this feature is full-body and does not depend on specific joints.

### Body tilt

Most of the research exploring tilt or angularity within body motion or positioning has focused on the head. There is compelling evidence to suggest that a downward or forward-tilted orientation of the head is uniquely associated with the portrayal of sadness (Crane & Gross, 2007; Masuda et al., 2010; Shafir et al., 2016; Wallbott, 1998), and these works suggest other patterns that may emerge. Wallbott (1998) suggests that having the head oriented backwards is associated with elated joy, raised shoulders are associated with both elated joy and hot anger, and raised arms are associated with each of the following: elated joy, cold anger, hot anger, and terror. Masuda et al. (2010) found that reclined posture is associated with pleasure, and that a mixture of reclined and straight posture is associated with relaxation. Shafir and colleagues (2016), on the other hand, found that a reclined tilt to the upper body is associated with fear. For the EMOKINE framework, we computed the head tilt with regards to the back (Section "Head tilt with respect to back") and with regards to the vertical axis (Section "Head tilt with respect to vertical").

#### Head tilt with respect to back

For the computation of this kinematic feature, we consider the three-dimensional positions for three keypoints: T8 vertebra, neck and head, dubbed here ${p}_{a}\left(t\right)$, ${p}_{b}\left(t\right)$, and ${p}_{c}\left(t\right),$ respectively. Then, we define the unit vectors going from T8 to the neck, and from neck to head, as:

$${u}_{ab}\left(t\right)=\frac{{p}_{b}\left(t\right)-{p}_{a}\left(t\right)}{\parallel {p}_{b}\left(t\right)-{p}_{a}\left(t\right){\parallel }_{2}}$$

$${u}_{bc}\left(t\right)=\frac{{p}_{c}\left(t\right)-{p}_{b}\left(t\right)}{\parallel {p}_{c}\left(t\right)-{p}_{b}\left(t\right){\parallel }_{2}}$$

Then, the head tilt with respect to the back $\mathrm{\alpha }\left(t\right)$ is the angle between ${u}_{ab}\left(t\right)$ and ${u}_{bc}\left(t\right)$, which can be computed as: $\alpha \left(t\right)=co{s}^{-1}\left({u}_{ab}{\left(t\right)}^{\mathrm{\top }}{u}_{bc}\left(t\right)\right)$, in radians, since the dot product between 2 unit vectors yields their cosine. This cosine is always non-negative, since we do not expect an angle larger than 90 degrees. We provide the average and MAD of $\alpha \left(t\right)$ across time.

#### Head tilt with respect to vertical

This feature is similar to the head tilt with respect to the back, but instead we measure the angle between ${u}_{bc}\left(t\right)$ and the global vertical:

$${u}_{\uparrow }\left(t\right)=\left(\begin{array}{c}0\\ 0\\ 1\end{array}\right)$$

This yields our desired feature, in radians: $\beta \left(t\right)=co{s}^{-1}\left({u}_{\uparrow }{\left(t\right)}^{\mathrm{\top }}{u}_{bc}\left(t\right)\right)$. We also provide the average and MAD as aggregation statistics.

### Space

The kinematic features in relation to space, in the context of this paper, refer to the physical area used by the dancer when performing the movement sequences. Previous research exploring space in this way as a kinematic parameter suggests that large travel distances (i.e., using a high proportion of the movement space) is associated with joy, and low travel distances (i.e., using a low proportion of the space) is associated with sadness (Sawada et al., 2003). It has also been suggested that movement in a variety of directions (i.e., a wider range in the movement space used) is associated with anger (Masuda et al., 2010). As measures of space within the EMOKINE framework, we provide the convex hull 2D (Section "Convex hull 3D") and 3D (Section "Convex hull 2D").

#### Convex hull 3D

Given the ${p}_{j}\left(t\right)$ locations for all body keypoints $\mathcal{J}$ at a given time $t$, the convex hull is the smallest convex envelope that contains all points. For example, if a person is extending their arms and legs in a t-pose and we take a frontal image of their keypoints, the corresponding 2D convex hull would be a convex polygon going from the head, to the hands, then from each hand to its respective foot, and then connecting the feet. In 3D, it follows the same principle but it also takes depth into account, yielding a convex polytope, where the vertices are the ${p}_{j}\left(t\right)$ keypoint positions. The convex hull can be used as a proxy for how much space is the person effectively occupying.

Formally, given the set of all keypoints $\mathcal{J}$, the 3D convex hull can be defined as the set (Boyd & Vandenberghe, 2004):

$${\mathcal{C}}_{3\mathcal{D}}\left(t\right)=\left\{\sum\limits_{j}{\theta }_{j}{p}_{j}\left(t\right) | j\in \mathcal{J},\theta \ge 0, \sum\limits_{j}{\theta }_{j}=1\right\}$$

And the ${c}_{3D}\left(t\right)$ feature we provide is the volume of ${\mathcal{C}}_{3\mathcal{D}}\left(t\right)$, in cubic meters ${m}^{3}$ (since the MVNX input positions ${p}_{j}\left(t\right)$ are given in meters). We used the SciPy Python library to compute this feature. Apart from the average and MAD aggregated statistics, we provide the following 2 aggregations:

• Global convex hull ${c}_{3D}\left(1,\dots ,{T}_{s}\right)$: This is the convex hull obtained from all points in a given sequence (as opposed to a specific timepoint), i.e., it covers all the locations where any keypoints has been at any time.

• Union of convex hulls ${\bigcup }_{t=1}^{{T}_{s}}{c}_{3D}\left(t\right)$: The main difference with the global convex hull, is that the union of convex hulls is a subset of the global convex hull, and is not necessarily convex: if the dancer jumps from the bottom left corner of the screen to the bottom right corner, the bottom center of the screen is part of the global convex hull, but not of the union of convex hulls. The reason is that there is no single timepoint where the bottom center is being covered, but if we consider all timepoints at once, we need to connect the left and right corners through the bottom center, thus making it part of the global convex hull.

#### Convex hull 2D

We compute this feature analogously to the 3D convex hull described before. The difference for this version is that we use the CamPos two-dimensional coordinates as source for this feature, i.e., two-dimensional vectors ${\uprho }_{j}\left(t\right)\in {\left[\mathrm{0,1}\right]}^{2}$ given in coordinates relative to the camera, going from $\left(\mathrm{0,0}\right)$ for the bottom-left corner to $\left(\mathrm{1,1}\right)$ for the top-right corner (i.e., it is a dimensionless ratio). The resulting convex hull is then a convex polygon (Boyd & Vandenberghe, 2004):

$${\mathcal{C}}_{2\mathcal{D}}\left(t\right)=\left\{{\sum }_{j}{\theta }_{j}{\rho }_{j}\left(t\right) | j\in \mathcal{J},\theta \ge 0, {\sum }_{j}{\theta }_{j}=1\right\}$$

And the computed feature ${c}_{2D}\left(t\right)$ is the surface of this polygon. Since the horizontal and vertical CamPos coordinates go from 0 to 1, the result is itself a (dimensionless) ratio between 0 and 1, telling how much of the total screen is covered by the convex hull of the dancer.

As with ${c}_{2D}\left(t\right)$, we provide four aggregations: average, MAD, global convex hull and union of convex hulls. We compute the surface of the 2D convex hull using the Python Shapely library.

## Validation of kinematic features and results of observer experiments

A series of validations of our kinematic and observer data are provided in this section. First, we provide a series of illustrations of the distributions of our kinematic features across the stimuli of the dataset. This is to compare the alignment of the stimuli between each other, to ensure that they are equivalent (e.g., between different visual presentations), and that differences emerge, where differences were expected (e.g., between different intended emotional expressions) (Section "Computational tests"). Second, we provide statistical tests of the observer ratings. In particular, we test that emotion recognition is above chance level (i.e., that the stimuli transmit the emotions that they were intended to transmit by the dancer), for all visual presentations, and we confirm that the beauty ratings vary as a function of the intended emotions by the dancer (Section "Results of observer experiments").

### Computational tests

We here provide a series of illustrations of computational validations, using either silhouette-dependent images or the keypoint-dependent data (that we retrieved from the XSENS® sensors) from our stimulus set. We use:

- foreground statistics to show that stimuli are balanced and within frames (Section "Foreground statistics").
- qualitative histograms (silhouettes) to show that stimuli are aligned with each other (Section "Qualitative histograms (silhouette-dependent kinematics)").
- kinematic histograms to show that the stimuli and features yield meaningful signal, not random noise (Section "Qualitative histograms (keypoint-dependent kinematics)").

#### Foreground statistics

**Fig. 5:**
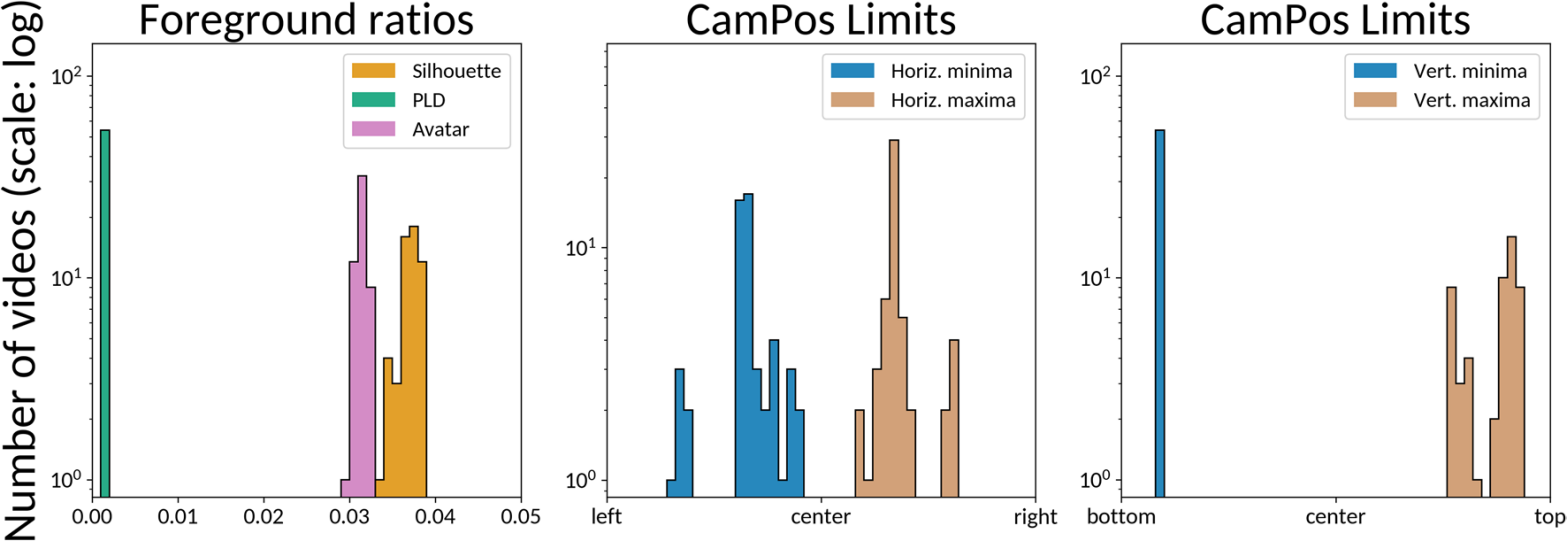
**Foreground statistics**. *Note*: Illustrations of the foreground statistics of all videos together (each unit on the *y*-axis is one video). We see a quite homogenous distribution of the dancer (= the foreground) across videos. **Foreground ratios**. This statistic indicates the ratio of the foreground with regards to the background. We see a concentrated distribution which indicates that there is not a big variation across stimuli of this set. **Camera positions (CamPos) for horizontal minima and maxima**. This figure answers the question of whether the dancer moves ‘out’ of the frame of the video horizontally, i.e., to the sides (*x*-axis). We see a quite symmetrical distribution around the center of the videos, and to the sides, with a very slight bias to the left. **Camera positions (CamPos) for vertical minima and maxima**. We see that the dancer occupies the vertical space quite homogeneously (*x*-axis). The high values are due to the arm movements up over the head, apart from that all camera positions are roughly at the same height

The foreground statistics describe the distribution of space occupied by the dancer (= the foreground) across videos. Using the silhouette images of the stimuli, we see a very homogenous distribution of the foreground throughout all videos for our three metrics (foreground ratios, and camera position limits for horizontal and vertical distributions). These results indicate that the videos of the stimuli set are equivalent in terms of foreground distribution (see Fig. 5 for illustration and a short description of the findings).

#### Qualitative histograms (silhouette-dependent kinematics)

**Fig. 6:**
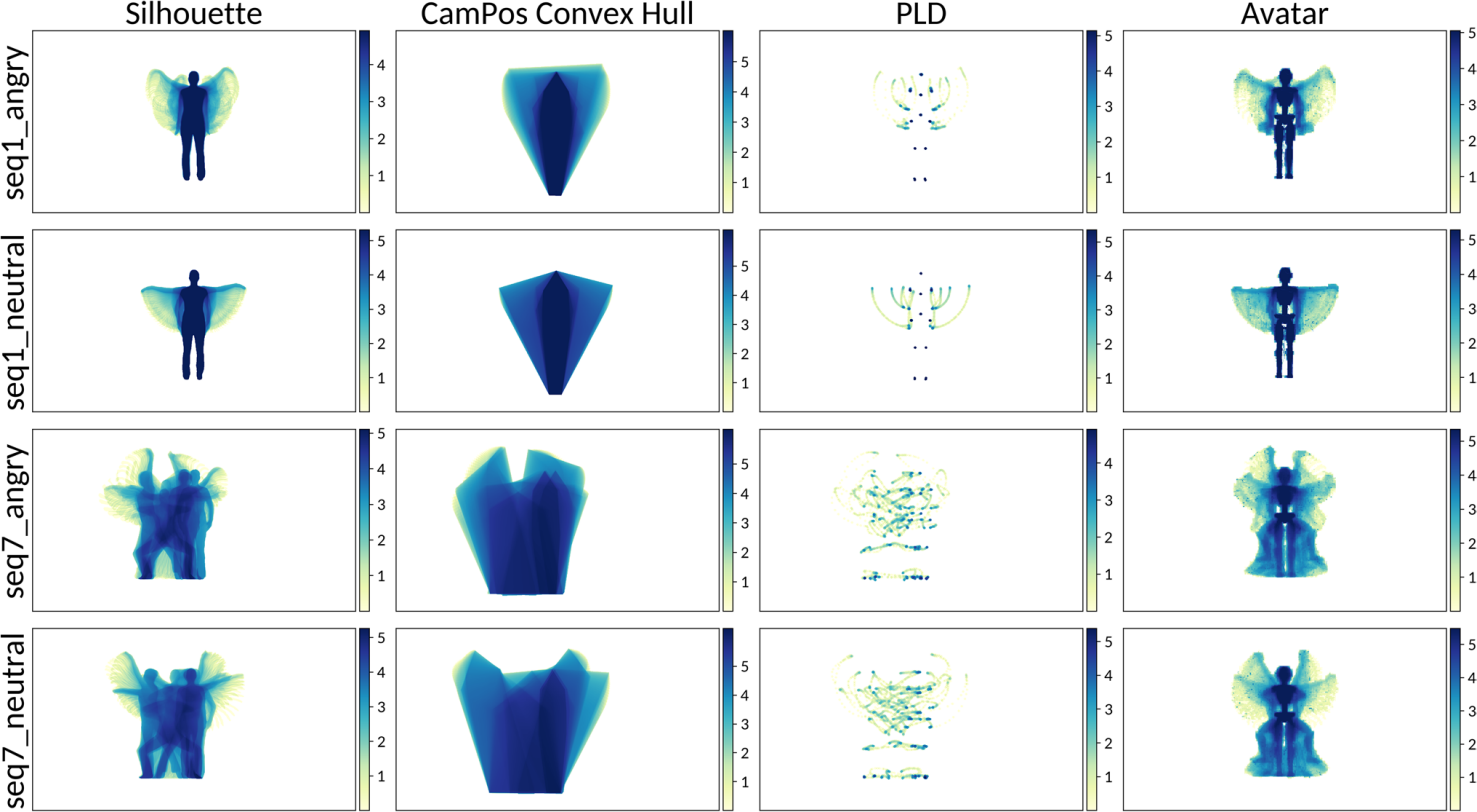
**Histograms of kinematics that depend on silhouette images**. *Note*: Frequencies of frames occupied by the dancer in space. Figure shows two illustrative stimuli as examples, sequence 1 (perfect alignment) and sequence 7 (good alignment except for the avatar stimulus). The illustrations for all 54 stimuli are in the appendix of the paper. The computation of the frequencies of frames occupied for the stimuli **silhouettes**, **convex hull**, **point-light displays (PLDs)**, and **avatar** stimuli depend on silhouette images. Silhouettes of the stimuli and CamPos (camera position) of the convex hull are calculated with the coordinates of the PLD (in Blender). The column with the avatar is from the XSENS® software. Across the columns, we see that the distribution of a single video is very equal among different modalities of videos, which indicates a very good alignment. It makes sense, since PLDs, convex hull and silhouettes are all from the same source, and they clearly show the same distribution. The silhouette occupies most space, then convex hull, avatar, PLDs. However, the illustration also shows a defect of the avatar (extracted from XSENS® software – the software is automatically adjusting the camera position. In videos 7–9 the dancer turns the upper body, which is corrected by the software so the camera position remains frontal throughout. This results in the very symmetric histograms in that column, while the other columns show movement also to the sides. As long as no turns are in the movement, the four stimuli modalities are aligned in terms of the frequencies of frames occupied by the dancer in space. There are no units on the axes because this is the horizontal and vertical space that the dancer occupies. The scale on the right side of the histograms shows the log of the frequency (color = log frequency of frames; 4 = frequency is 100 (np.log(freq)) = 4.61). The log scale allows seeing more differentiation the distribution of frames occupied by the dancer in space

We use qualitative histograms (silhouette images of the videos) to show that the stimuli are aligned with each other. We computed the frequencies of frames occupied for the silhouettes, convex hull, point-light displays (PLDs), and (D) the avatar stimuli, which all depend on silhouette images. The Silhouette occupies most space, then convex hull, avatar, PLDs. The histograms show a nice alignment across all four modalities. However, they also show a defect of the avatar (extracted from XSENS® software) – the software is automatically adjusting the camera position. In videos 7–9, the dancer turns the upper body, which is corrected by the software so the camera position remains frontal throughout. This results in the very symmetric histograms in that column, while the other columns show movement also to the sides. This means that as long as no turns are in the movement, the four modalities are aligned in terms of the frequencies of frames occupied by the dancer in space (see Fig. 6 for illustration and a short description of the findings for two of the nine sequences; sequence 1 and 7). The illustrations for the remaining sequences are in the appendix of the paper (see Figs. 15, 16, 17, 18, 19).

**Fig. 7:**
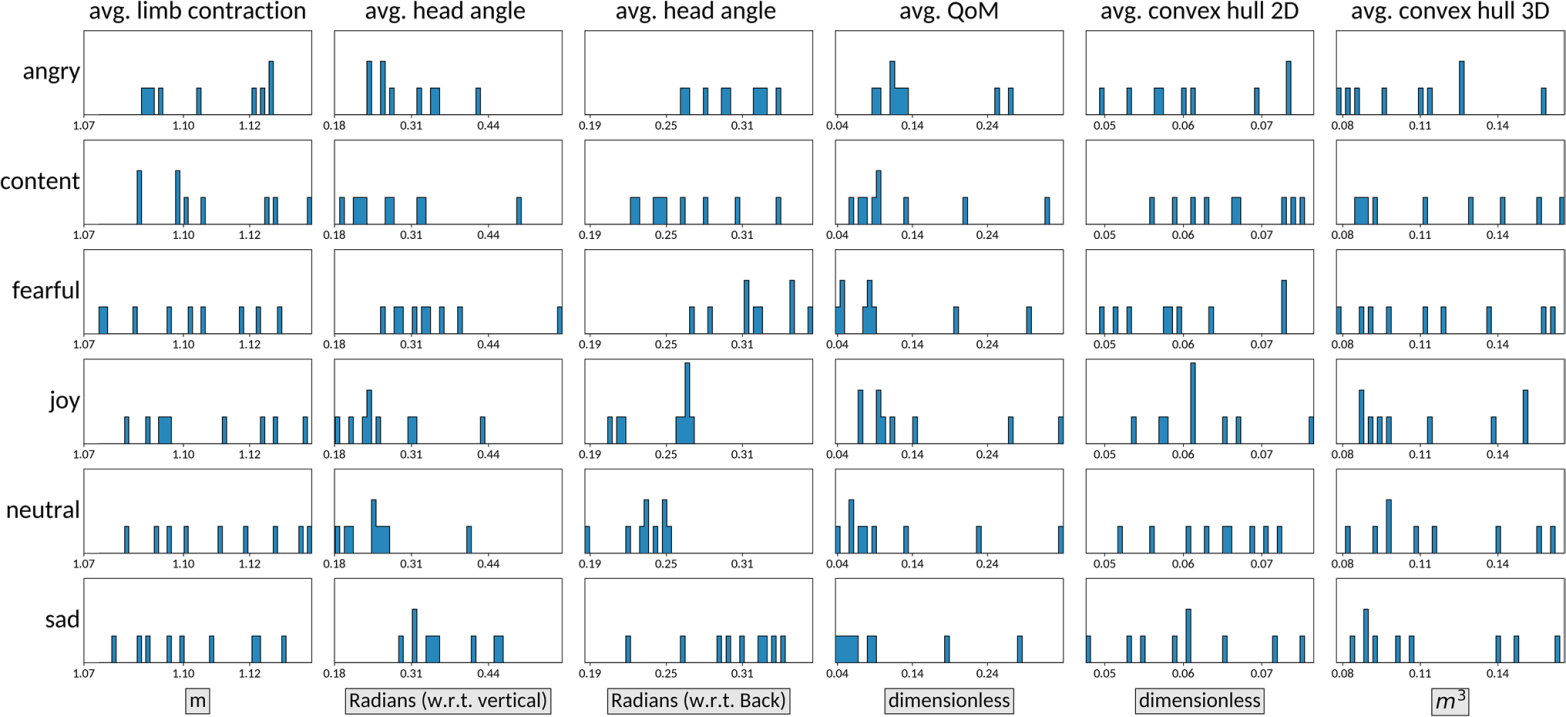
**Kinematics that depend on silhouette images as a function of emotion**. Note: Qualitative histograms illustrating the distributions of the silhouette-dependent kinematics **average limb contraction, average head angle with respect to vertical, average head angle with respect to back, average quantity of motion (QoM), mean convex hull 2D, and mean convex hull 3D**. The kinematics are plotted separately for each emotion (angry, content, fearful, happy, neutral, sad) across all videos. Comparing the distribution of each kinematic feature across the different emotions (column wise, top to bottom), we observe differences in the distributions, as a function of intended emotion. For example, the distribution of the mean head angle for angry videos (top graph) is left skewed, while it is right skewed for sad videos (bottom graph). The *y*-axis shows number of videos. The *x*-axis is the scale of the respective kinematic (see label on the bottom of each column)

We use another series of qualitative histograms to explore visually the kinematics of average limb contraction, mean head angle (with respect to vertical and back), average quantity of motion (QoM), mean convex hull 2D and mean convex hull 3D. We plotted these separately for each emotion (angry, content, fearful, happy, neutral, sad), across all videos (see Fig. 7 for illustration and a short description of the findings).

#### Qualitative histograms (keypoint-dependent kinematics)

**Fig. 8:**
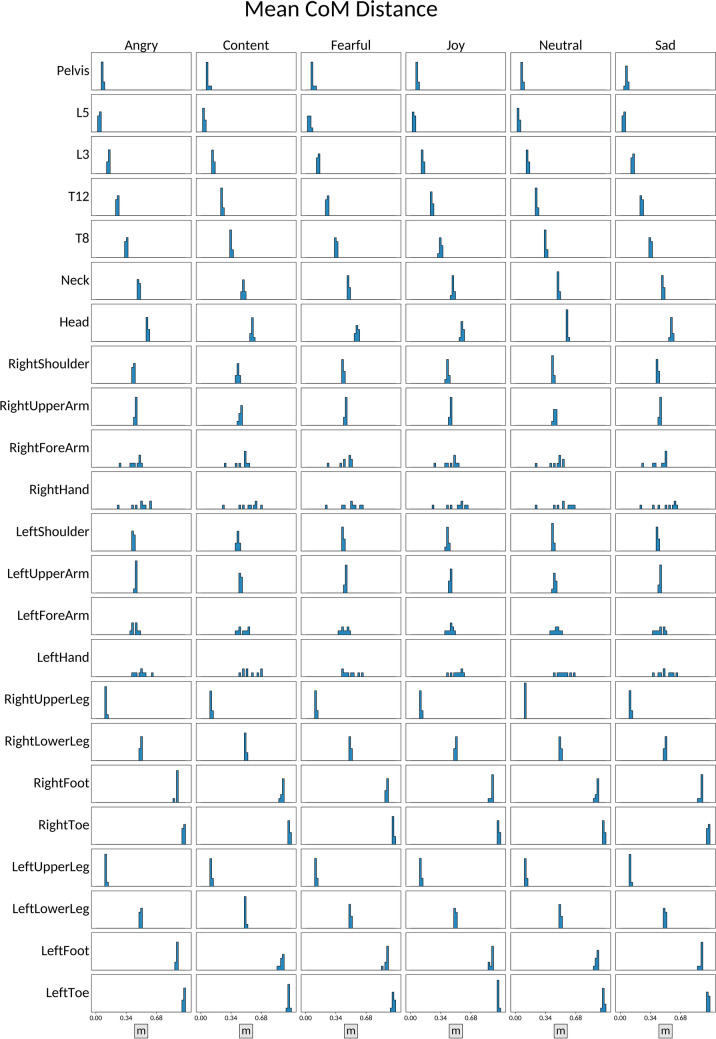
Distance to the center of mass. *Note*: Distance from center of mass (CoM). Histograms for each of the 23 keypoints given by the XSENS® system, across the six emotions (**angry, content, fearful, joy, neutral, sad**). The further away from the center of mass the keypoint is (pelvis = very close; hands = very far), the distribution changes. We also see that the legs remain relatively stable throughout, which is in accordance with the choreographies set out in Table 2 above: the movements were mostly confined to the arms, with little leg movement. We also observe that the distributions vary across emotions, especially for the arms, which again is in accordance with the intention of the dancer during stimuli creation, where the intention was to confine the expressivity mostly to the arms

**Fig. 9:**
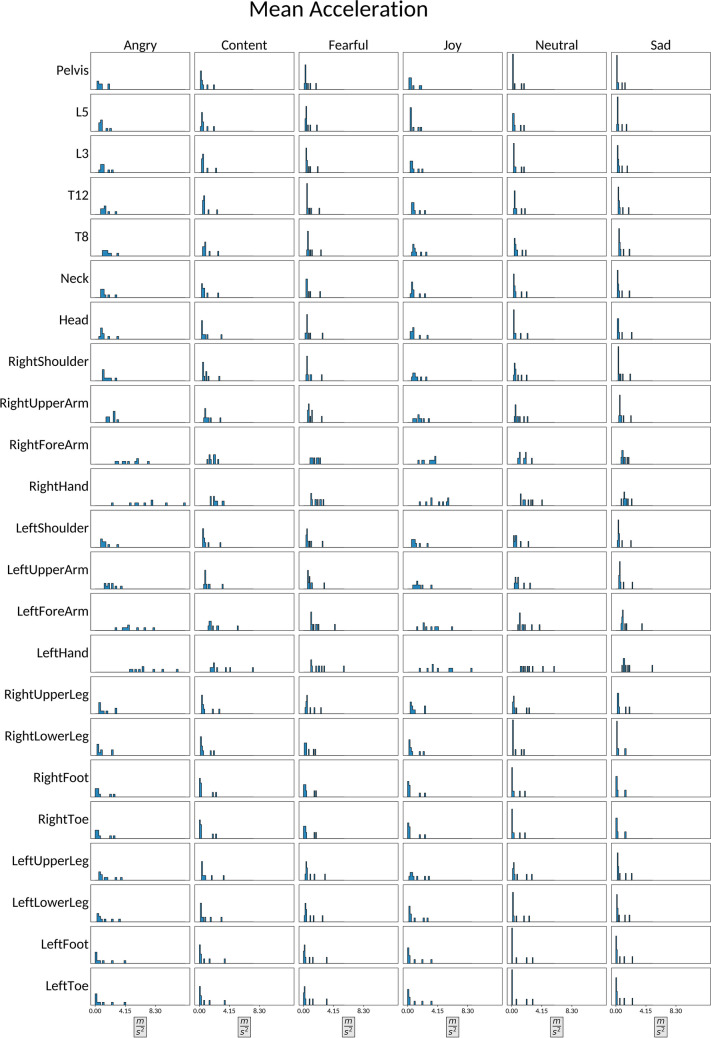
Mean acceleration. *Note*: Mean acceleration. Histograms for each of the 23 keypoints given by the XSENS® system, across the six emotions (**angry, content, fearful, joy, neutral, sad**). Acceleration changes are evident across emotions especially for arms and pelvis and the head. The legs remain relatively stable throughout, which is in accordance with the choreographies set out in Table 2: the movements were mostly confined to the arms, with little leg movement

For the keypoint-dependent kinematics (retrieved from the XSENS® sensors), we provide illustrations for the distance to the center of mass (Fig. 8) and average acceleration (Fig. 9). The figures show histograms for each of the 23 keypoints across the six emotions (angry, content, fearful, joy, neutral, sad). The distance to center of mass figure shows that the further away from the center of mass the keypoint is (pelvis = very close; hands = very far), the distribution changes. We also see that the legs remain relatively stable throughout, which is in accordance with the choreographies set out in Table 2 above: the movements were mostly confined to the arms, with little leg movement. Hence, these distributions confirm the choreographies. We also observe that the distributions vary across emotions especially for the arms, which again is in accordance with the intention of the dancer during stimuli creation, where the intention was to confine the expressivity mostly to the arms (see Figs. 8 and 9 for illustrations and a short description of the findings).

The remaining keypoint-dependent kinematics are provided in the appendix; for dimensionless jerk, see Fig. 20, for angular acceleration, see Fig. 21, and for angular velocity, see Fig. 22.

### Results of observer experiments

As described in Sections "Participants’ online experiment" and "Procedure for Human Observer Experiment", human observers performed an emotion recognition task and an aesthetic judgment task on all pilot stimuli. We here provide a technical test to ensure that:

- Intended emotional expression was recognized above chance level (Sections "Chance level analysis: Visual presentation", "Chance Level Analysis: Emotion Category", and "Chance Level Analysis: Visual Presentation x Emotion Category").
- Beauty ratings depended on the intended emotional expression of the dancer (Section "Observer experiment beauty ratings").

#### Chance level analysis: Visual presentation

Chi-square *t* tests were used to determine whether observer recognition rates were above chance for the four visual presentations (four levels; avatars, full-light displays (FLDs), point-light displays (PLDs), silhouettes; chance level: 100%/6 emotion categories = 16.67%). Results showed that the stimuli of all four visual presentations had been recognized above chance level (all *p*s < .001).

**Fig. 10:**
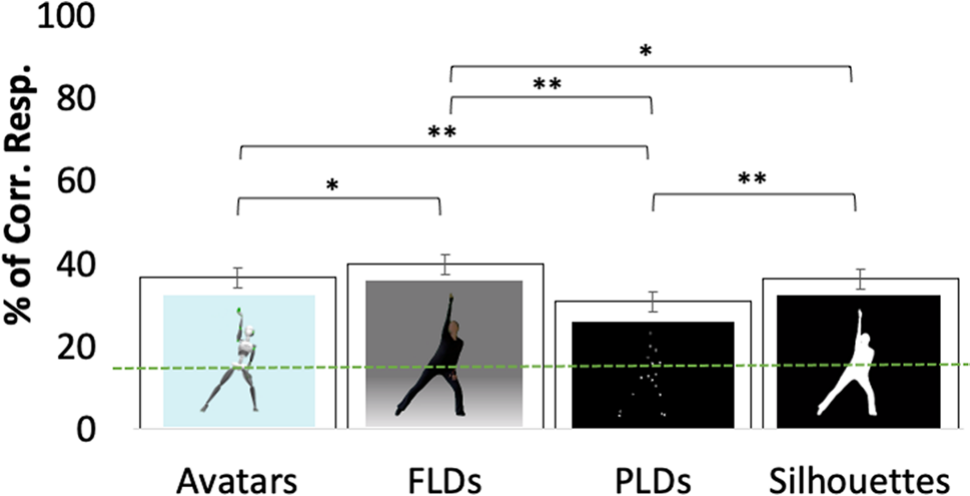
Emotion recognition rate: Main effect of visual presentation. *Note*: Observer emotion recognition scores as a function of visual presentation was above chance level (16.67%) for all four visual presentations (*dotted line* illustrates chance level). Observer emotion recognition was highest for FLDs than for all other visual presentations, while PLDs had the lowest observer emotion recognition rates. Observer recognition rates for avatars and silhouettes were higher than recognition rates of PLDs. Observer recognition rates for avatars and silhouettes did not differ between each other. ** *p* > .001. * *p* > .02. *Bars* show averages; *error bars* represent SE. *Dotted line* represents emotion recognition chance level at 16.5%. FLD = full-light displays; PLDs = point-light displays

To explore whether there was a difference between the four visual presentations in terms of emotion recognition accuracy, we performed a 1 x 4 RM ANOVA with the factor *visual presentation* (four levels; avatars, FLDs, PLDs, silhouettes), and the dependent variable *percent of correct responses* (‘correct responses’ = when observers guessed the emotion that the dancer was intending while performing the movements). There was a main effect of *visual presentation* (F(3,393) = 21.352, *p* < .001, partial *η*^*2*^ = .140). Estimated marginal means showed that FLD videos were recognized best (EMM = 39.82%; SE = 1.12), followed by avatar videos (EMM = 36.45%; SE = .98), then silhouette videos (EMM = 36.20%; SE = 1.10), and finally PLDs (EMM = 30.77%; SE = .98). Bonferroni corrected pair-wise comparisons showed that some of these differences were significant. FLDs were recognized above all others (all *p*s > .018). Avatars and silhouettes were recognized equally well (*p* = 1.00), and emotions expressed in the PLDs were recognized below all other visual presentations (all *p*s > .001). These results are illustrated in Fig. 10.

#### Chance level analysis: Emotion category

Chi-square *t* tests were used to determine whether observer recognition rates were above chance for the six emotion categories (chance level: 100% / 6 emotion categories = 16.67%). The stimuli of the emotion categories anger, content, joy, neutral, sad (regardless of visual presentation) had been recognized above chance level (all *p*s > .014), while fear had not (*p* = .645).

**Fig. 11:**
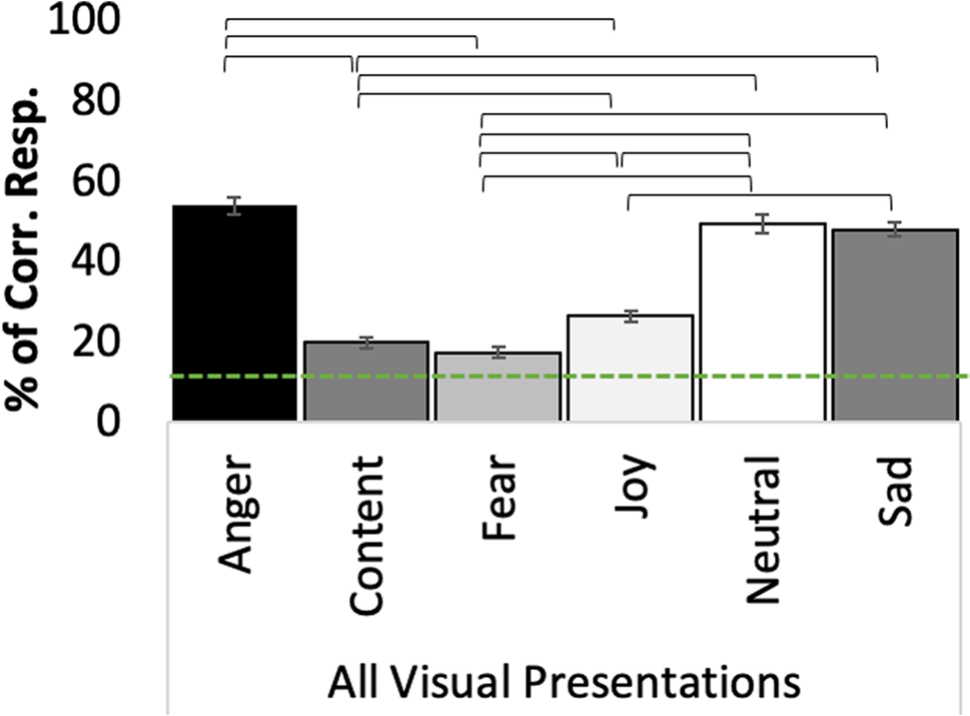
Emotion recognition rate: Main effect of emotion. *Note*: Observer emotion recognition scores as a function of emotion (regardless of visual presentation) was above chance level (16.67%) for five of the emotions intended for the dancer (anger, content, joy, neutral, sad), but not for fear (*dotted line* illustrates chance level). Observer emotion recognition rates were highest for angry videos > neutral videos > sad videos > joyful video > content videos > fearful videos. There were significant differences between all pairs of emotions (all *p*s > .01), except between the pairs anger-neutral, content-fear, and sad-neutral. *Brackets* indicate significant differences, all *p* > .001, except the pair anger-sad, which was *p* = .015

To explore whether there were differences between recognition rates as a function of the different emotion categories intended by the dancer, we performed a 1x6 RM ANOVA with the factor *emotion category* (six levels; anger, content, fear, joy, neutral, sad), and the dependent variable *percent of correct responses* (‘correct responses’ = when observers guessed the intended emotion). There was a main effect of *emotion category* (F(5,655) = 99.457, *p* < .001, partial *η*^*2*^ = .432). The observer recognition rates were highest for angry videos (EMM = 53.91%; SE = 1.98) > neutral videos (EMM = 49.432%; SE = 2.17) > sad videos (EMM = 48.04%; SE = 1.86) > joyful video (EMM = 26.39%; SE = 1.32) > content videos (EMM = 19.76; SE = 1.24) > fearful videos (EMM = 17.30%; SE = 1.36). Bonferroni corrected pairwise comparisons showed that recognition rates for angry, neutral and sad stimuli were highest and did not differ between each other (all *p*s < .227) but differed significantly from all other emotions (all *p*s > .001). Further, emotion recognition rates for joyful videos was higher than for fearful videos (*p* > .001). All comparisons are illustrated in Fig. 11.

#### Chance level analysis: Visual presentation x emotion category

**Fig. 12:**
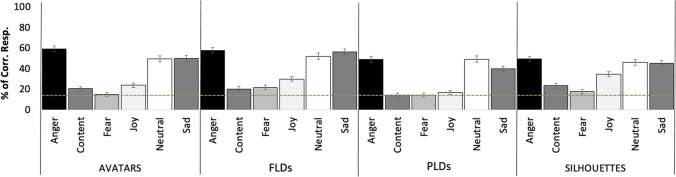
Visual presentation x emotion for percentage of correct responses. *Note*: Illustrating observer emotion recognition rates for the different emotions between the different visual presentations, for all emotion categories. For the avatars, all emotions were recognized above chance (all *p*s > .050), except for fear (*p* = .363). For the FLDs, all emotions were recognized above chance (all *p*s > .016), except for content (*p* = .071). For the PLDs, emotions were recognized above chance (all *p*s > .001), except for content (*p* = .226), fear (*p* = .315) and joy (*p* = .898). For the silhouettes, emotions were recognized above chance (all *p*s > .050), except for fear (*p* = .643). *Bars* show emotion averages; *dotted line* illustrates chance level; *error bars* represent SE. *FLD* full-light displays; *PLDs* point-light displays

For each visual presentation, Chi-square *t* tests were used to compare emotion recognition rates for each emotion against the chance level of 16.67%. For the avatars, all emotions were recognized above chance (all *p*s > .050), except for fear (*p* = .363). For the FLDs, all emotions were recognized above chance (all *p*s > .016), except for content (*p* = .071). For the PLDs, emotions were recognized above chance (all *p*s > .001), except for content (*p* = .226), fear (*p* = .315), and joy (*p* = .898). For the silhouettes, emotions were recognized above chance (all *p*s > .050), except for fear (*p* = .643). For an overview of the results see Fig. 12.

#### Confusion matrices emotion ratings

Four confusion matrices were computed, one for each visual presentation. They represent the observers’ emotion judgments as a function of intended and decoded emotion. The advantage of confusion matrices is that the ‘confused’ responses (i.e., the wrong emotion judgments) for a stimulus can be compared across all emotion categories at a glance (see Banse & Scherer, 1996; Scherer & Scherer, (2011), for a detailed explanation). These matrices are set in Tables 3 and 4 for avatars, in Table 5 for FLDs, in Table 6 for PLDs and in Table 7 for silhouettes.

**Table 4:**
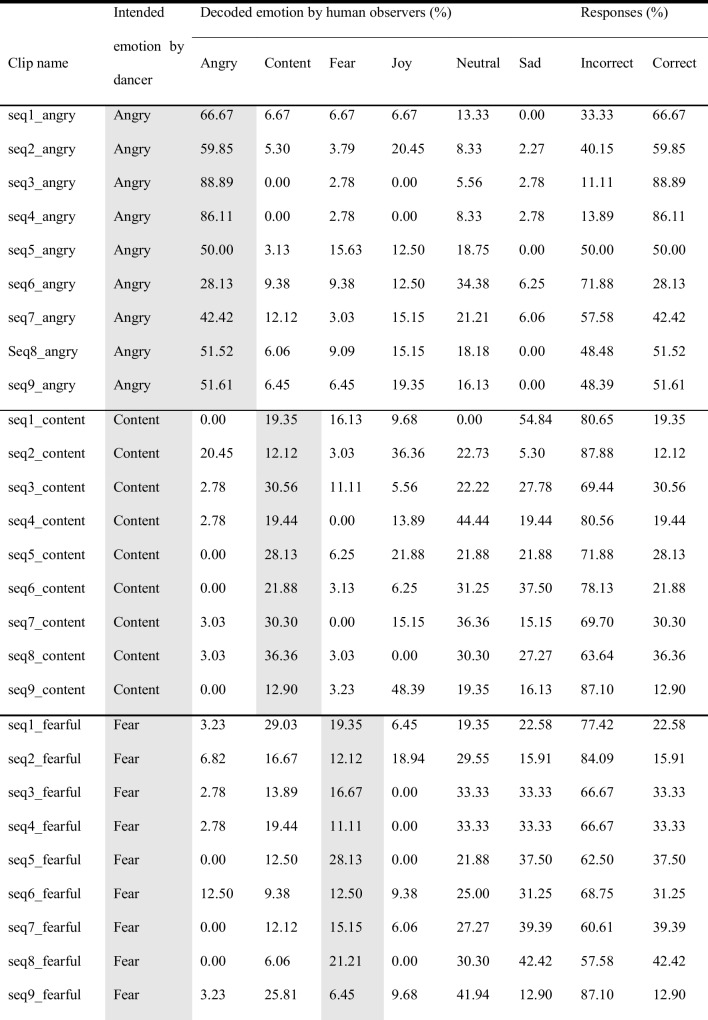

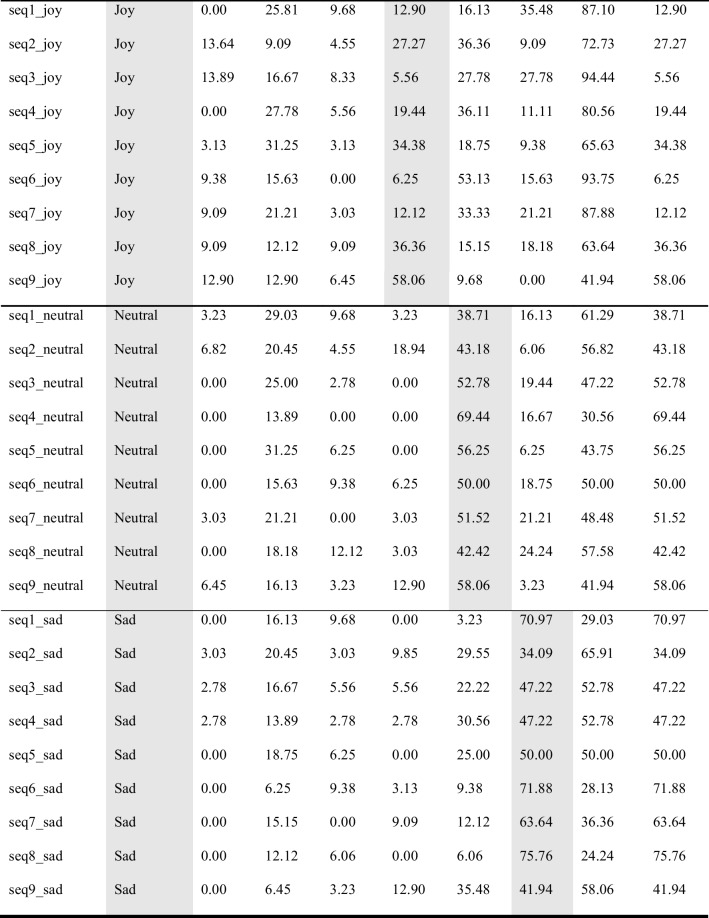
Confusion matrix: EMOKINE avatar stimuli

Avatar stimuli. Percent of responses as a function of intended emotion of 54 avatar stimuli. *Left side columns* indicate clip name and the intended emotional expression by the dancer when performing the movement (“Intended Emotion by dancer”). The percentages of emotion judgments made by the human observers in the experiment are represented in each of the columns under the label “Decoded emotion”. The confusion matrix illustrates the distribution of the given emotion judgment for each of the different emotion options. The *grey shading* indicates when intended and decoded emotion correspond. The *two columns on the right* set out the total incorrect and correct responses for each clip (i.e., when the intended and the decoded emotion where the same -correct-, or not -incorrect-)

**Table 5:**
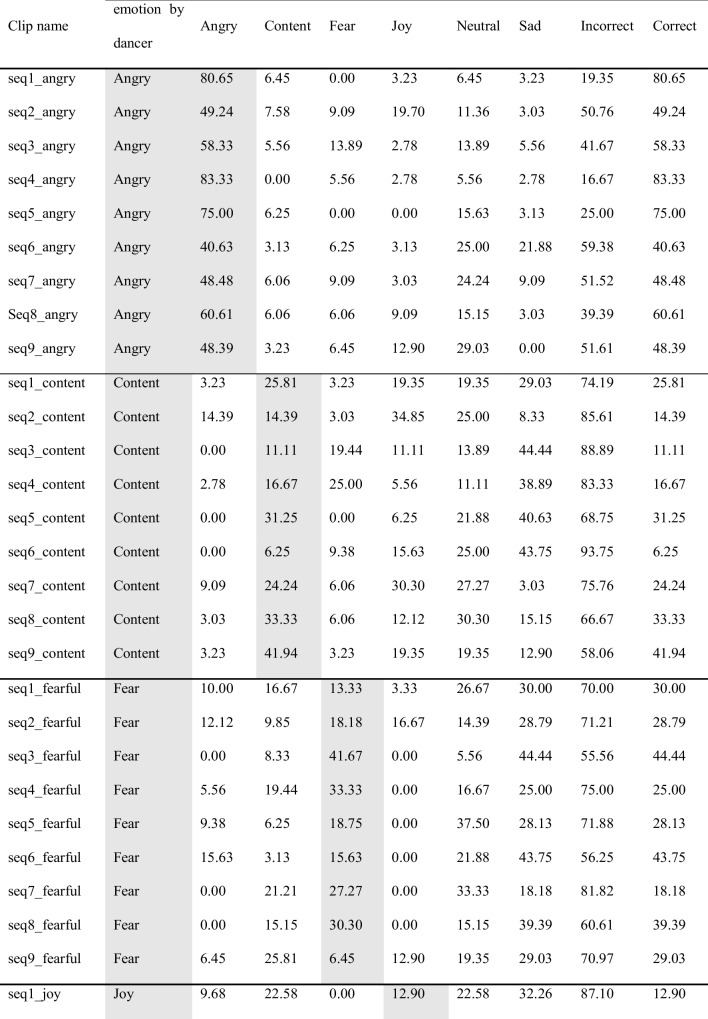

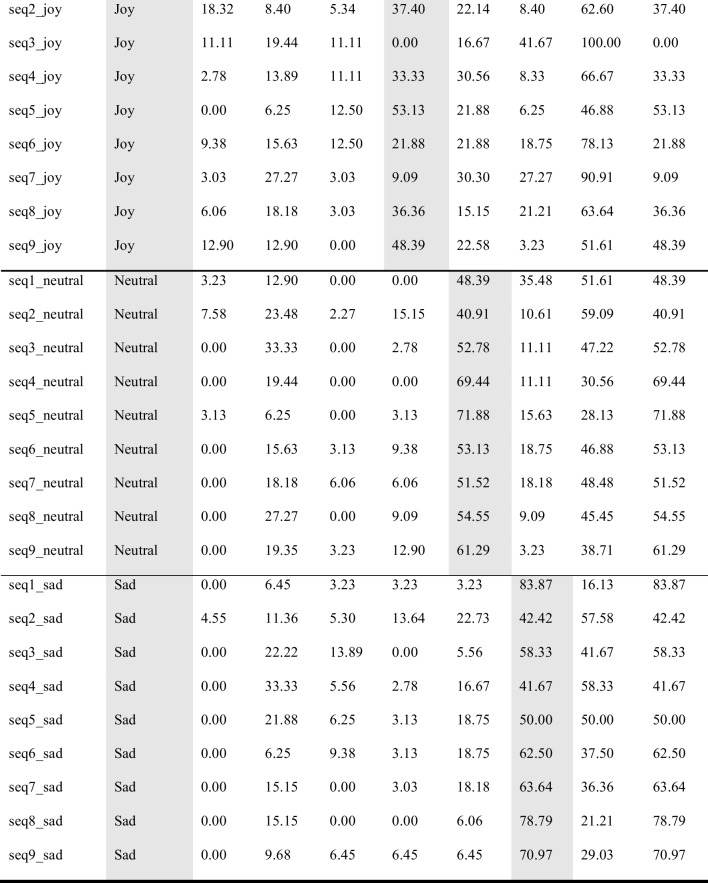
Confusion matrix: EMOKINE full-light displays (FLDs) stimuli

Full-light displays (FLDs) stimuli. Percent of responses as a function of intended emotion of 54 FLD stimuli. *Left side columns* indicate clip name and the intended emotional expression by the dancer when performing the movement (“Intended emotion by dancer”). The percentages of emotion judgments made by the human observers in the experiment are represented in each of the columns under the label “Decoded emotion”. The confusion matrix illustrates the distribution of the given emotion judgment for each of the different emotion options. The grey shading indicates when intended and decoded emotion correspond. The *two columns on the right* set out the total incorrect and correct responses for each clip (i.e., when the intended and the decoded emotion where the same -correct-, or not -incorrect-)

**Table 6:**
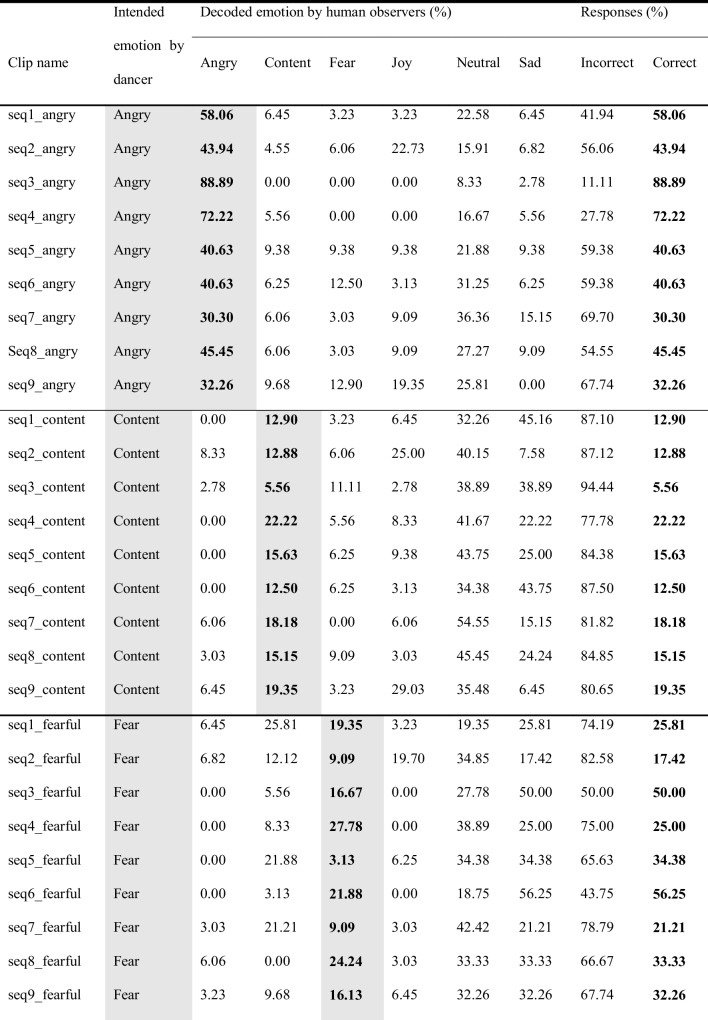

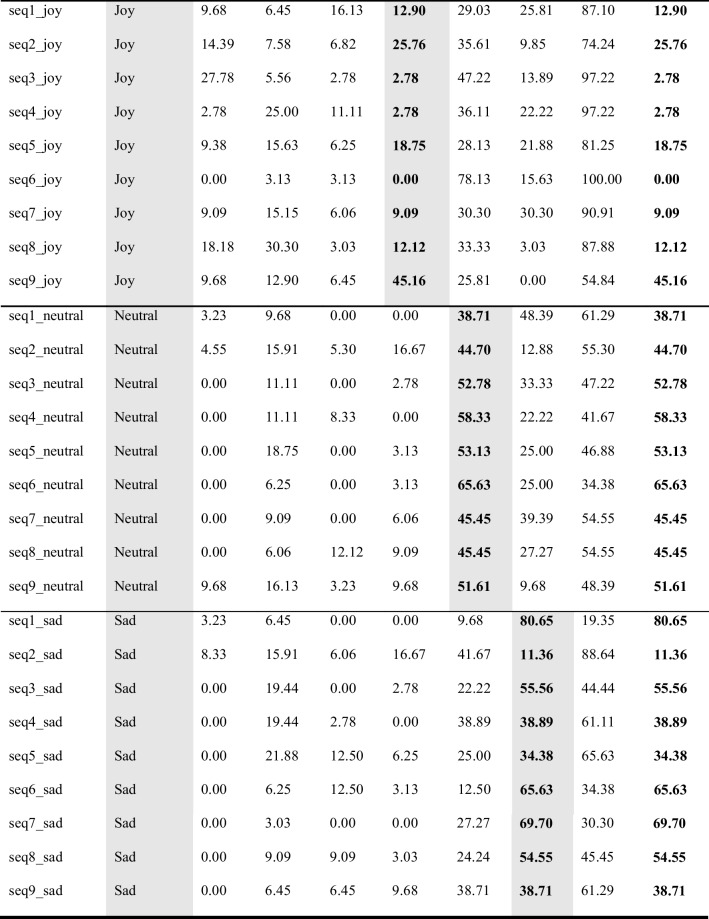
Confusion matrix: EMOKINE point-light displays (PLDs) stimuli

Point-light display (PLD) stimuli. Percent of responses as a function of intended emotion of 54 PLD stimuli. *Left side columns* indicate clip name and the intended emotional expression by the dancer when performing the movement (“Intended emotion by dancer”). The percentages of emotion judgments made by the human observers in the experiment are represented in each of the columns under the label “Decoded emotion”. The confusion matrix illustrates the distribution of the given emotion judgment for each of the different emotion options. The *grey shading* indicates when intended and decoded emotion correspond. The *two columns on the right* set out the total incorrect and correct responses for each clip (i.e., when the intended and the decoded emotion where the same -correct-, or not -incorrect-)

**Table 7:**
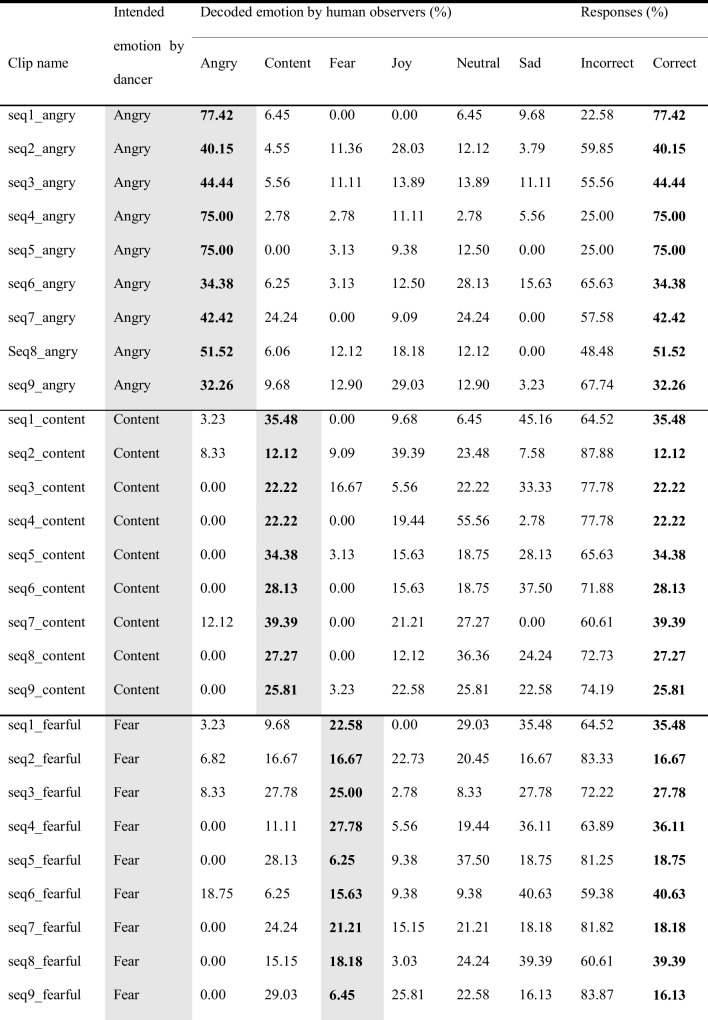

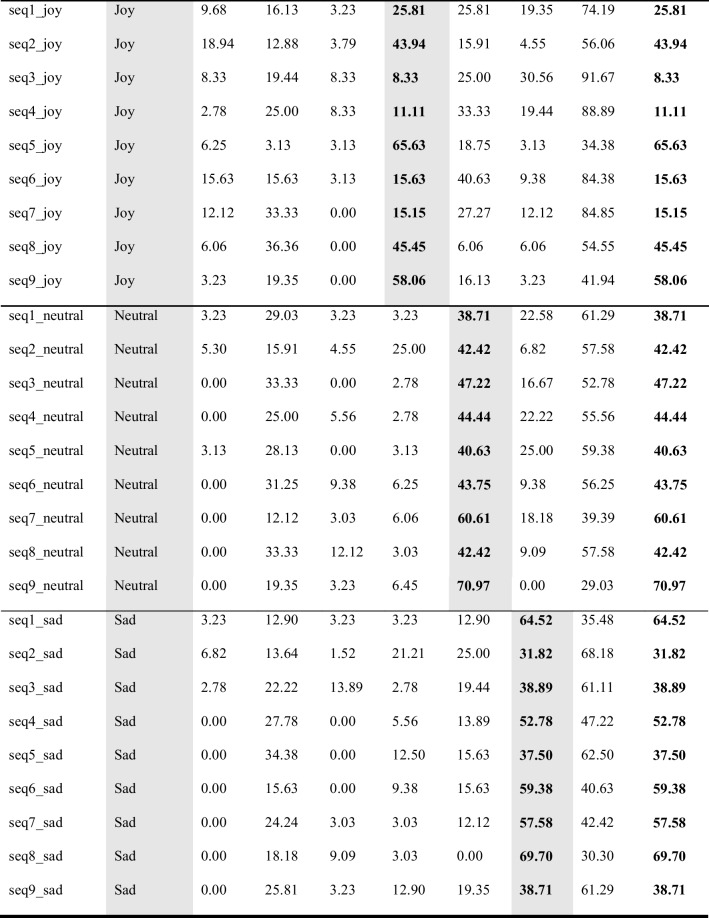
Confusion matrix: EMOKINE silhouette stimuli

Silhouette stimuli. Percent of responses as a function of intended emotion of 54 silhouette stimuli. *Left side columns* indicate clip name and the intended emotional expression by the dancer when performing the movement (“Intended emotion by dancer”). The percentages of emotion judgments made by the human observers in the experiment are represented in each of the columns under the label “Decoded emotion”. The confusion matrix illustrates the distribution of the given emotion judgment for each of the different emotion options. The *grey shading* indicates when intended and decoded emotion correspond. The *two columns on the right set* out the total incorrect and correct responses for each clip (i.e., when the intended and the decoded emotion where the same -correct-, or not -incorrect-)

#### Observer experiment beauty ratings

**Fig. 13:**
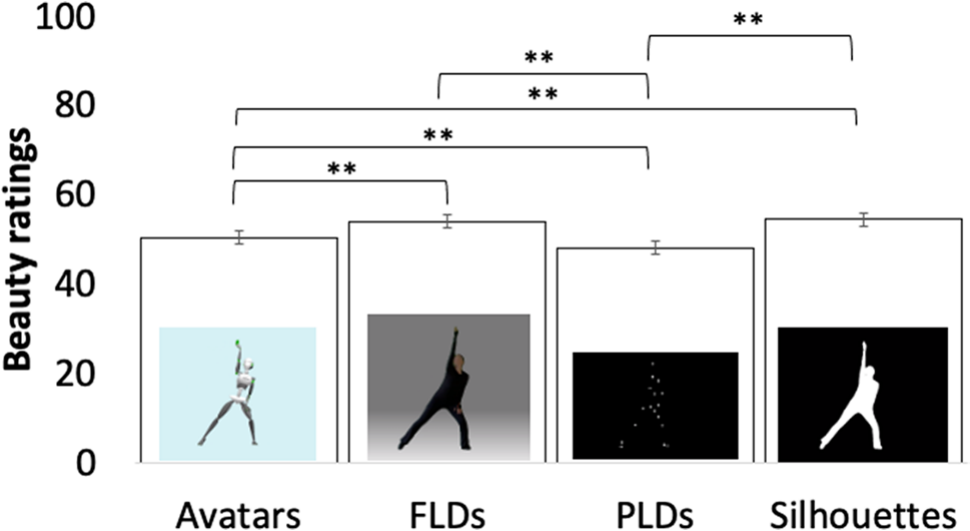
Beauty ratings: Main effect of visual presentation. *Note*: Observer beauty ratings as a function of visual presentation from 0 (not beautiful) to 100 (very beautiful). Observer beauty ratings were highest for silhouette stimuli followed by FLDs that did not differ between each other (*p* = .280), followed by avatar stimuli, and, finally, PLDs which all differed significantly between each other (all *p*s > .001). *Bars* show averages; *error bars* represent SE. *FLD* full-light displays, *PLDs* point-light displays

A 1 × 4 RM ANOVA was conducted with the factor *visual presentation* (four levels; avatars, full-light displays (FLDs), point-light displays (PLDs), silhouettes). The dependent variable was ‘*Beauty rating*’ on a scale from 0 (not beautiful) to 100 (very beautiful). There was a main effect of *visual presentation* (F(3,393) = 35.336, *p* < .001, partial *η*^*2*^ = .212). Estimated marginal means (EMM) showed that silhouette stimuli were rated as most beautiful (EMM = 54.35; SE = 1.33), followed by FLD stimuli (EMM = 53.91; SE = 1.33), then avatar stimuli (EMM = 50.17; SE = 1.34), and, finally, PLDs (EMM = 47.93; SE = .93). Bonferroni corrected pair-wise comparisons showed that FLDs and silhouette beauty ratings did not differ significantly between each other (*p* = .280), but all other comparisons were significant (all *p*s > .001). FLDs and silhouettes being rated as more beautiful than avatars and PLDs, PLDs being rated the least beautiful (see Fig. 13).

**Fig. 14:**
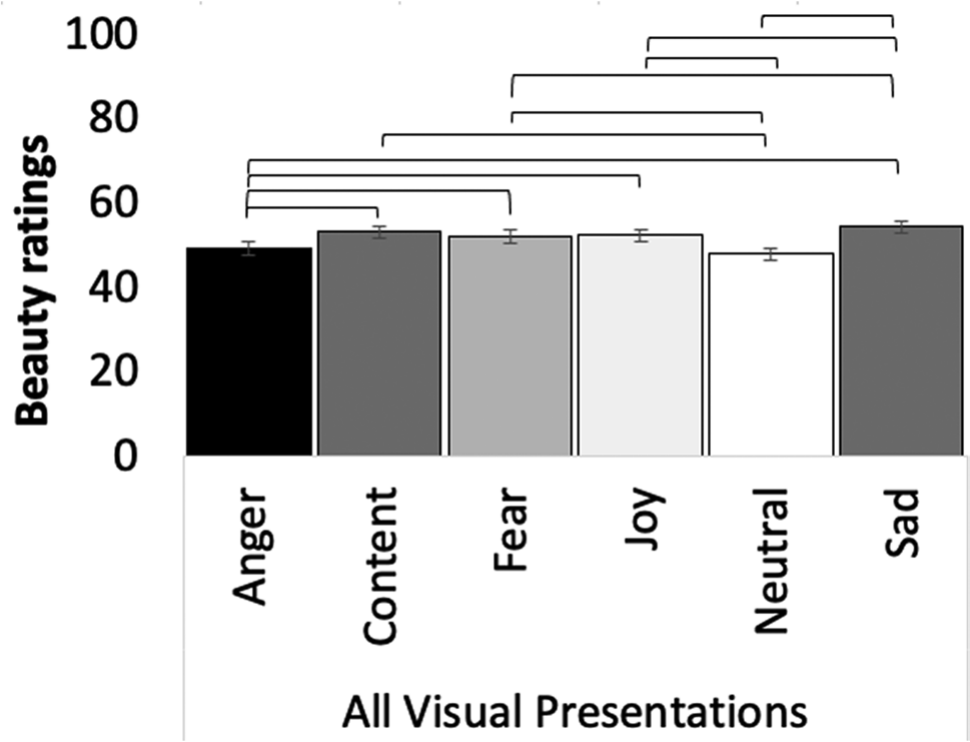
Beauty ratings: Main effect of emotion. *Note*: The *bars* show observer beauty rating averages across *visual presentations* (0; 100) were highest for sad stimuli (EMM = 54.46; SE = 1.33) > content stimuli (EMM = 53.23; SE = 1.31) > joyful stimuli (EMM = 52.46; SE = 1.30) > fearful stimuli (EMM = 52.10; SE = 1.33) > anger stimuli (EMM = 49.32; SE = 1.31) > neutral stimuli (EMM = 48.01; SE = 1.31). *Brackets* show significant differences in beauty ratings between emotion categories, all *p*s > .001, except for the comparison joy-sad, which was p = .017 (Bonferroni corrected). Error bars represent SE

**Fig. 15:**
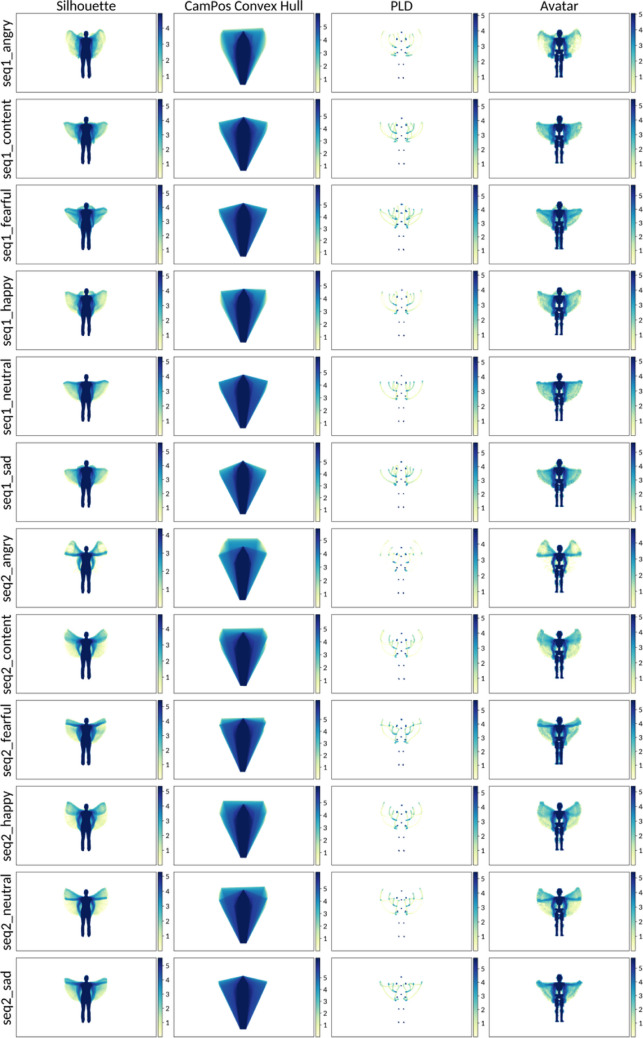
Histograms of kinematics that depend on silhouette images (Sequence 1–2)

**Fig. 16:**
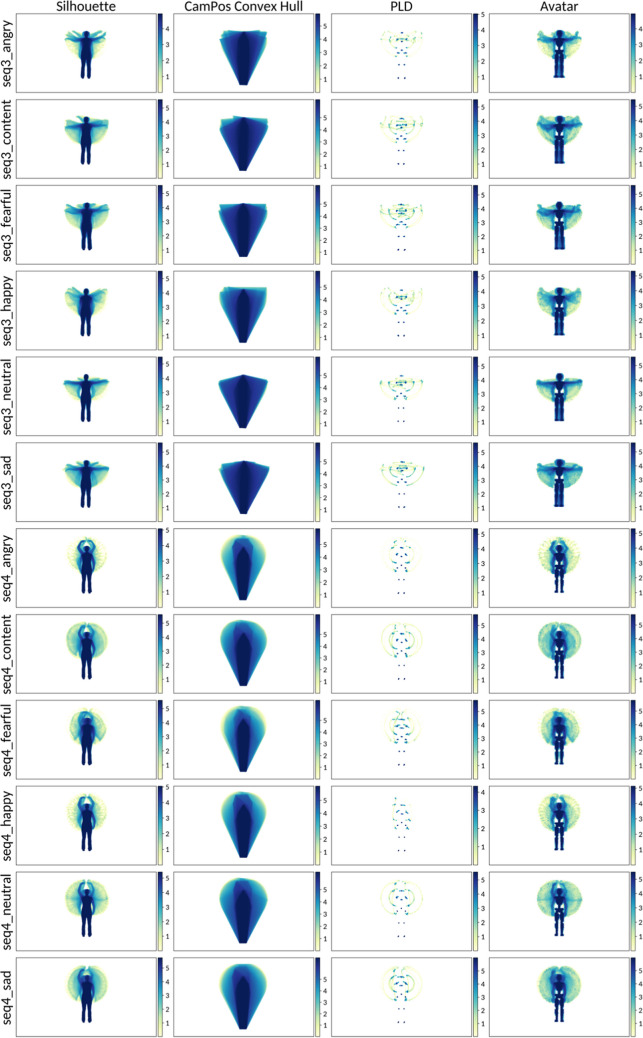
Histograms of kinematics that depend on silhouette images (Sequence 3–4)

**Fig. 17:**
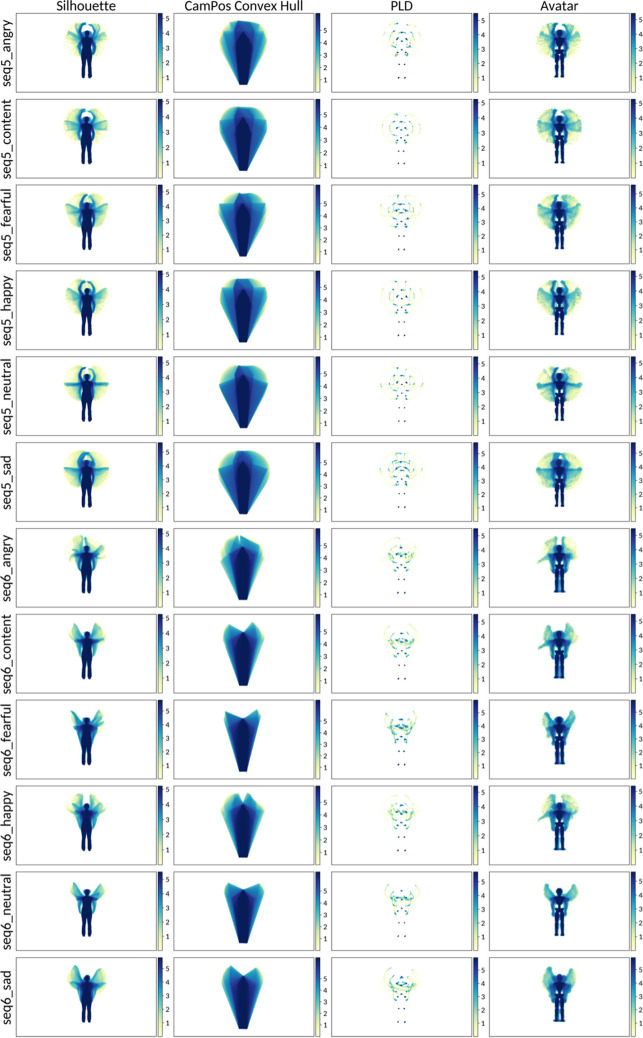
Histograms of kinematics that depend on silhouette images (Sequence 5–6)

**Fig. 18:**
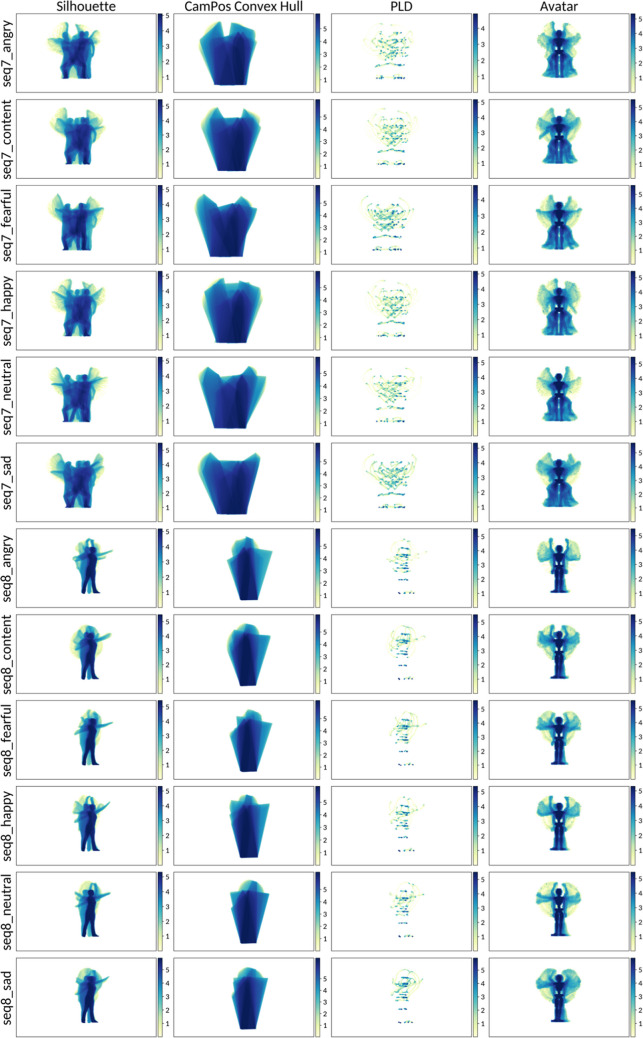
Histograms of kinematics that depend on silhouette images (Sequence 7–8)

**Fig. 19:**
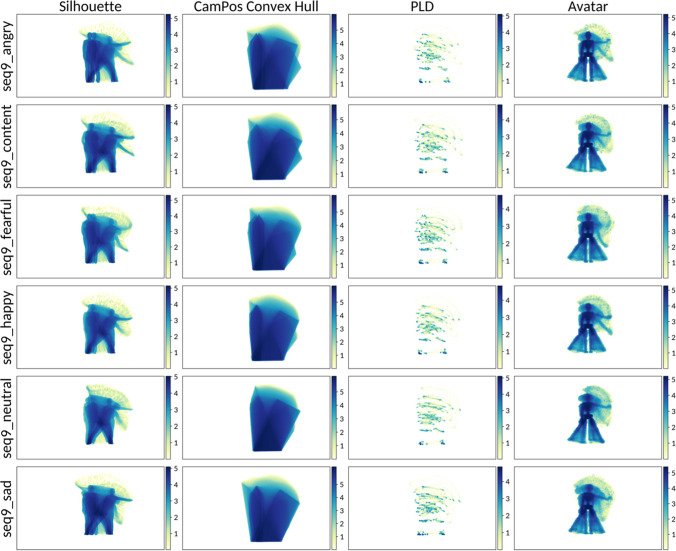
Histograms of kinematics that depend on silhouette images (Sequence 9)

**Fig. 20:**
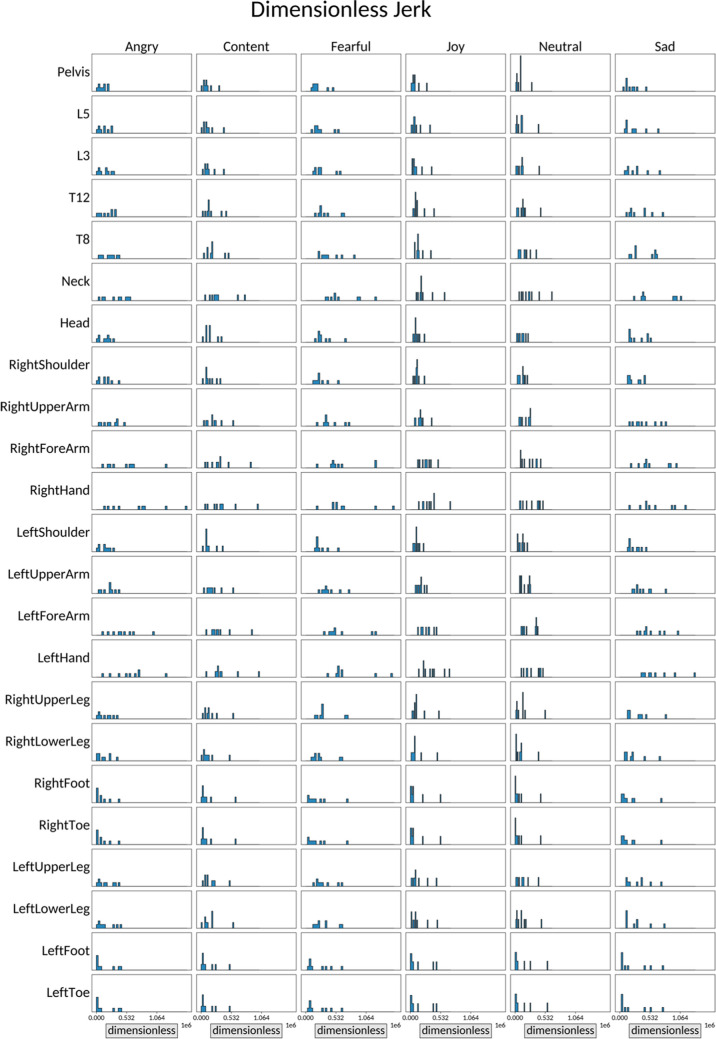
Dimensionless jerk

**Fig. 21:**
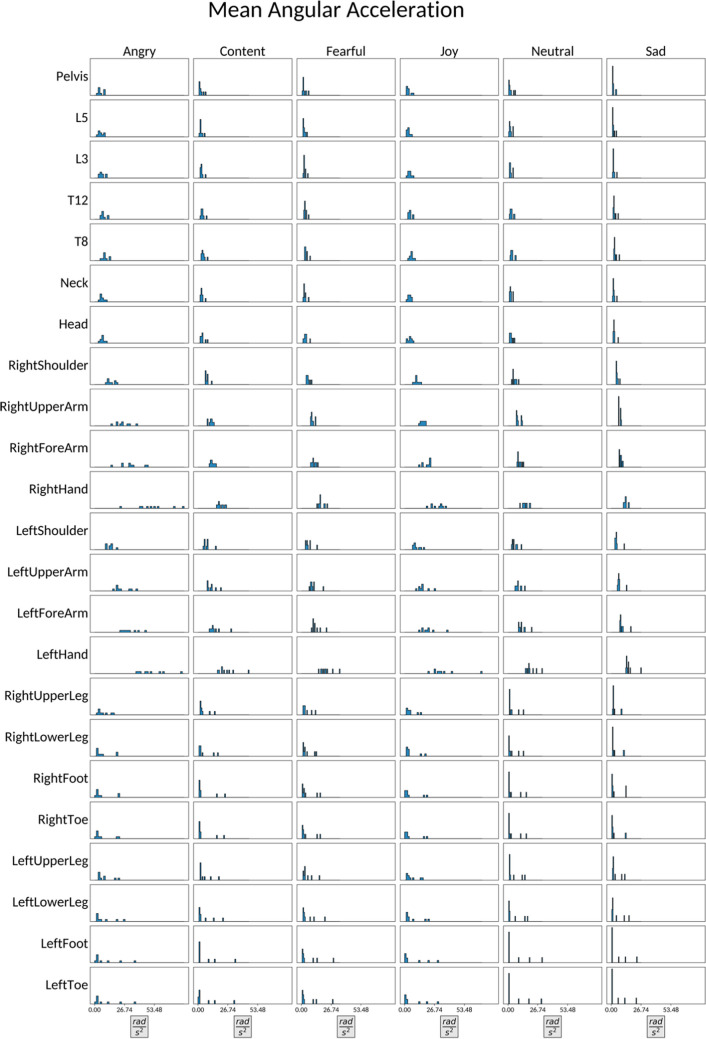
Angular acceleration

**Fig. 22:**
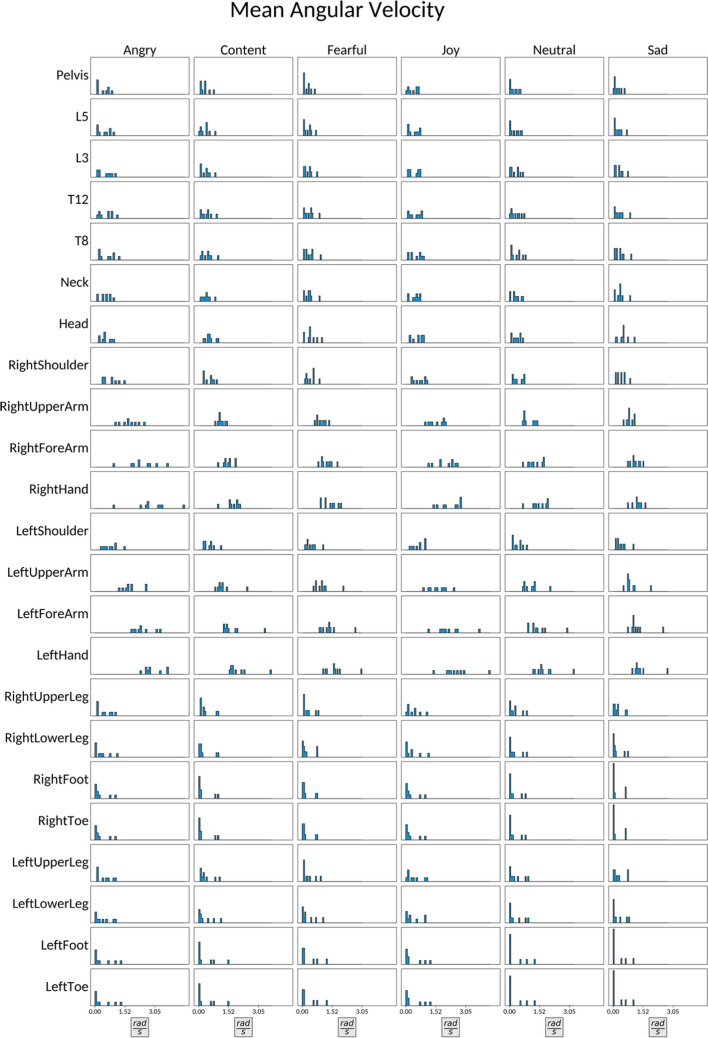
Angular velocity

A 1 × 4 RM ANOVA was conducted with the factor *emotion* (six levels; anger, content, fear, joy, neutral, sad). The dependent variable was ‘*beauty rating*’ on a scale from 0 (not beautiful) to 100 (very beautiful). There was a main effect of *emotion* (F(5,655) = 32.562, *p* < .001, partial *η*^*2*^ = .199). Descriptively, the observers’ beauty ratings were highest for sad stimuli (EMM = 54.46; SE = 1.33) > content stimuli (EMM = 53.23; SE = 1.31) > joyful stimuli (EMM = 52.46; SE = 1.30) > fearful stimuli (EMM = 52.10; SE = 1.33) > anger stimuli (EMM = 49.32; SE = 1.31) > neutral stimuli (EMM = 48.01; SE = 1.31). Bonferroni corrected pair-wise comparisons showed that there were significant differences in beauty ratings between most categories (all *p*s > .001, except for the comparison joy-sad, which was *p* = .017; Bonferroni corrected). There was *no* significant difference between anger and neutral, nor between fear and joy. Also, the beauty ratings to the emotion contentment did not differ from those for fear, joy, and sadness (see Fig. 14 for an illustration of these results).

## Discussion and conclusion

We provide the EMOKINE software, computational framework, and pilot dataset of emotional movement for research in experimental psychology, affective neuroscience and computer vision. The key contribution of the project to the wider community is a computational framework comprising a detailed plan, software, and code for creation of highly controlled emotional body movement datasets at scale in the future. Comprehensive procedure instructions and kinematic feature extraction code are provided via releases on GitHub. The pilot dataset and its renderings into four different visual presentations (avatars, full-light-displays, point-light-displays, and silhouettes), along with observer ratings and the kinematic data are available on Zenodo under a Creative Commons Attribution 4.0 International License.

A series of computational validations and an observer experiment confirmed the validity of the EMOKINE pilot dataset and the creation procedure. Besides these validations provided here, the dataset has been shown to be useful to assess research questions in health psychology, for example, about how dance breaks during work hours may improve mood and motivation (Schmidt et al., 2023). Datasets created following the EMOKINE suite may be particularly useful for addressing questions about which kinematic features drive high emotion recognition and/or misclassifications. Yet for future large scale experiments, we remind researchers that the EMOKINE pilot dataset was only created with a single dancer and only contains nine movement sequences. For generalizability and scaling up, creating datasets with several dancers as models and more sequences would be advisable. 

Foreground statistics showed that stimuli were balanced and within frames, qualitative histograms (silhouettes) confirmed that stimuli were aligned with each other, kinematic histograms indicated that the stimuli and features yielded meaningful signals, not random noise.

The observer experiment confirmed that emotional expression was recognized above chance level in the pilot dataset. Emotion recognition was highest for full-light displays (FLDs), than for all other visual presentations, while point-light displays (PLDs) had the lowest emotion recognition rates. Observer recognition rates for avatars and silhouettes were higher than recognition rates of PLDs. Observer recognition rates for avatars and silhouettes did not differ between each other. With regards to the emotion categories, observer emotion recognition rates were highest for angry videos (> neutral videos > sad videos > joyful video > content videos > fearful videos). We present confusion matrices, one for each visual presentation, which represent the observers’ emotion judgments as a function of intended and decoded emotions. Confusion matrices allows to compare ‘confused’ responses (i.e., the wrong emotion judgments) for a stimulus across all emotion categories at a glance, following previous work in the field (Scherer & Scherer, 2011). With regards to aesthetic judgment, observer beauty ratings were highest for silhouette stimuli followed by FLDs, followed by avatar stimuli, and, finally, PLDs. Furthermore, aesthetic judgment was highest for sad stimuli (> content stimuli > joyful stimuli > fearful stimuli > anger stimuli > neutral stimuli).

Pilot stimuli intended to express fear were hardest to recognize for observers, and average recognition across fearful stimuli was not above chance for any of the visual presentations except in the FLDs; an effect that has previously been reported with other stimuli sets (Atkinson et al., 2007; Camurri et al., 2003; Christensen et al., 2023; Christensen et al., major revisions; Dahl & Friberg, 2007; Pasch & Poppe, 2007; Smith & Cross, 2022). Contentment, the emotion added to this dataset was recognized above chance in the visual presentations avatar and silhouettes, but surprisingly not in FLDs and PLDs.

Importantly, as described in previous work, also in the EMOKINE pilot dataset, aesthetic judgment (i.e., beauty) differed significantly between all emotions. This adds another datapoint to previous findings that suggest that aesthetic judgment can be an implicit emotion recognition task (Christensen et al., 2019; Christensen et al., 2023; Christensen et al., major revisions).

Creating future datasets based on the procedure set out above has three main advantages. First, as shown with the EMOKINE pilot dataset creation procedure, we propose to use complex movements. A dancer repeated several choreographies six times each, maintaining the same movements, but expressing different emotional intentions at each repetition. Traditionally, emotional ‘actions’ are often used in emotion datasets (e.g., jumping of joy or recoiling in fear), which makes the emotion rather obvious. For EMOKINE, the dancer used exactly the same dance choreography to express six different emotional intentions, thus, increasing the usefulness of the dataset to assess subtle kinematic features in emotional movement that is not emotional actions. Second, the EMOKINE dataset creation procedure proposes to include more emotional intentions. Here, we included six emotional intentions, namely anger, contentment, fear, joy, neutrality, and sadness. Classically, datasets rarely contain the emotion contentment (Ekman, 1973/2015; Ekman & Friesen, 1971), which increases the usefulness of EMOKINE. ‘Contentment’ is another positively valenced emotion like joy, yet of low arousal; symmetrical to what anger (negative valence, high arousal) is to sad (negative valence, low arousal). Third, the EMOKINE software provides, for the first time, thirty-two statistics from twelve kinematic features that can be obtained from one same dataset, namely, speed, acceleration, angular speed, angular acceleration, limb contraction, distance to center of mass, quantity of motion, dimensionless jerk (integral), head angle (with regards to vertical axis and to back), and space (convex hull 2D and 3D). Average, median absolute deviation (MAD) and maximum value were computed for each.

Future iterations of the dataset creation plan may take into account that the four visual presentations were not parametrically varied, but could be, using the kinematic data to vary the visual presentation of the stimuli, and also to control the exact length of the stimuli. Further, the XSENS avatar rendering was not 100% overlapping with the other visual presentations because the positioning of the legs was not fixed by the software, causing the avatar to move slightly unnaturally with less common arm and leg movement combinations. Finally, we acknowledge the WEIRD focus of this dataset creation, and suggest exploring non-Western dance with the same procedure as e.g., Christensen et al. (major revisions).

The EMOKINE software and pilot dataset is the outcome of a proof-of-principle dataset creation procedure for highly controlled kinematic video datasets of emotionally expressive full-body movement sequences. The pilot data for EMOKINE was recorded via the XSENS® system, however, with small alterations that we have outlined above and on the GitHub repository, the software can be used with data obtained from other motion capture systems too. The novelty of EMOKINE lies in the successful integration of the experimental control requirements for psychology and affective neuroscience research involving human participants and, simultaneously, ensuring the technical intricacies required for datasets in computer vision and related fields.

## Data Availability

The dataset and all materials can be downloaded from Zenodo: https://zenodo.org/record/7821844 (Zenodo DOI: 10.5281/zenodo.7821844 ). The software is available on GitHub: https://github.com/andres-fr/emokine Comprehensive Readme files accompany the data and software. None of the experiments was preregistered.

## Appendix

This section contains several additional histograms from our Computational Tests Section "Computational tests".

See Figs. 15, 16, 17, 18, 19, 20, 21, 22.

## Declarations

### **Open practices statement**

The dataset and all materials can be downloaded from Zenodo: https://zenodo.org/record/7821844 (Zenodo 10.5281/zenodo.7821844). The software is available on GitHub: https://github.com/andres-fr/emokine Comprehensive Readme files accompany the data and software. None of the experiments was preregistered.
